# Cognitive Performance as a Zeitgeber: Cognitive Oscillators and Cholinergic Modulation of the SCN Entrain Circadian Rhythms

**DOI:** 10.1371/journal.pone.0056206

**Published:** 2013-02-18

**Authors:** Howard J. Gritton, Ashley M. Stasiak, Martin Sarter, Theresa M. Lee

**Affiliations:** 1 Department of Psychology, University of Michigan, Ann Arbor, Michigan, United States of America; 2 Neuroscience Program, University of Michigan, Ann Arbor, Michigan, United States of America; Vanderbilt University, United States of America

## Abstract

The suprachiasmatic nucleus (SCN) is the primary circadian pacemaker in mammals that can synchronize or entrain to environmental cues. Although light exerts powerful influences on SCN output, other non-photic stimuli can modulate the SCN as well. We recently demonstrated that daily performance of a cognitive task requiring sustained periods of *attentional effort* that relies upon basal forebrain (BF) cholinergic activity dramatically alters circadian rhythms in rats. In particular, normally nocturnal rats adopt a robust diurnal activity pattern that persists for several days in the absence of cognitive training. Although anatomical and pharmacological data from non-performing animals support a relationship between cholinergic signaling and circadian rhythms, little is known about how endogenous cholinergic signaling influences SCN function in behaving animals. Here we report that BF cholinergic projections to the SCN provide the principal signal allowing for the expression of cognitive entrainment in light-phase trained animals. We also reveal that oscillator(s) outside of the SCN drive cognitive entrainment as daily timed cognitive training robustly entrains SCN-lesioned arrhythmic animals. Ablation of the SCN, however, resulted in significant impairments in task acquisition, indicating that SCN-mediated timekeeping benefits new learning and cognitive performance. Taken together, we conclude that cognition entrains non-photic oscillators, and cholinergic signaling to the SCN serves as a temporal timestamp attenuating SCN photic-driven rhythms, thereby permitting cognitive demands to modulate behavior.

## Introduction

Circadian rhythms are shared by almost all organisms and provide a highly adaptive mechanism to anticipate daily environmental events. The suprachiasmatic nucleus of the hypothalamus (SCN) acts as the central pacemaker in mammals and is tightly synchronized to the external twenty-four hour period by the light-dark (LD) cycle or solar day [1]. Synchronization to the LD cycle can be eliminated by bilateral ablation of the SCN [2]. Animals and humans, however, can also synchronize, or entrain, to other non-photic environmental cues. Some non-photic stimuli, such as timed running wheel access and social interaction, exert their influences on behavior through phase resetting of clock genes within the SCN [3]–[4]. Other non-photic stimuli including fear learning, access to palatable foods, food restriction, and methamphetamine can produce entrainment even in SCN ablated animals [5]–[8]. Although oscillators outside the SCN are necessary to entrain to these cues, recent evidence suggests that non-SCN oscillators often interact or compete with the SCN to influence biological and behavioral rhythms [9]–[11].

We have recently reported that timed daily attentional performance produces a dramatic change in the daily activity profiles of nocturnal male Sprague-Dawley rats when trained during the light-phase. This includes a robust diurnal phenotype concomitant with a daily increase in anticipatory activity that occurs several hours before task onset and persists for a period of several cycles after training ceases under constant conditions [12]–[13]. Control experiments including daily handling, timed daily water access during the light-phase, or operant training did not produce similar phenotypes. In addition, we found that training on operant tasks with low attentional demand or spatial memory tasks during the light-phase failed to alter circadian markers of photic-driven entrainment. While all of the control treatments produced some level of anticipation, all treatment animals remained nocturnal with little or no anticipatory activity once the daily manipulation ceased [12]–[13].

Because changes in circadian architecture were limited to tasks requiring *attentional effort*, we speculated that the entraining effect of cognitive task performance may be mediated through neurotransmitter systems essential for attentional performance. Cholinergic signaling is normally augmented during tasks requiring sustained attention [14]–[15] and above chance performance on attentionally demanding tasks is dependent upon the integrity of the basal forebrain cholinergic system [16]. Furthermore, daily rises in acetylcholine levels associated with task performance become anticipatory and time-locked when training occurs at the same time every day and this precise daily increase in acetylcholine persists for several days in the absence of cognitive training [17]. Taken together, this data suggests that basal forebrain cholinergic output becomes synchronized to the time of daily cognitive performance.

Anatomical studies have established a direct connection between cholinergic projections originating from the basal forebrain neurons of the medial septum, nucleus basalis, diagonal band, substantia inominata, and the SCN [18]. The exact role of these projections is unknown, but studies suggest that they influence cellular and behavioral activity through modulatory effects on the SCN. For example, injections of carbachol, a nonspecific acetylcholine agonist, into the SCN induces subjective night phase advances and delays similar to those produced by light [19]–[20]. Likewise, *in vitro* application of carbachol to SCN brain slices phase advances the neural firing activity of SCN neurons, thereby modifying the time of day at which peak firing rates occur [21]. The SCN also undergoes cholinergic-signaling dependent changes in cholinergic receptor expression both immediately, and at intervals of 24 hours, for several continuous cycles in response to salient stimuli [22]–[23] revealing that cholinergic signaling may play a prominent role in SCN entrainment.

The present study examined how attention-demanding task performance interacts with circadian mechanisms in the SCN. We used site-specific injections of the immunotoxin 192 IgG-saporin to selectively eliminate cholinergic projections to the SCN originating from the basal forebrain, thereby testing the necessity of forebrain cholinergic neurotransmission in mediating entrainment to cognitive tasks. Next, we trained animals with bilateral SCN electrolytic lesions to determine if cognitive performance alone, in the absence of the SCN, is capable of organizing circadian activity as has been reported for some other non-photic entrainers, including food and methamphetamine [24]–[26].

Our findings reveal that daily timed cholinergic signaling in the SCN during the light-phase promotes diurnal behavior and entrainment through cognition. Attentional performance during the endogenous rest phase, however, also produced a profound state of internal desynchrony denoted by a discord between activity and temperature rhythms consistent with those described in chronic shift-workers. SCN ablation did not block the synchronized activity produced by cognitive training and suggests that entrainable oscillator(s) sensitive to cognitive performance exist independent of the SCN. Collectively these findings provide a basis for the hypothesis that *in vivo* cholinergic signaling, modulated by cognitive demand, entrains non-SCN oscillators and promotes diurnal activity through cholinergic-SCN interactions. This coordinated activity promotes the expression of cognition-entrained rhythms to facilitate daily fixed time learning and cognitive performance.

## Materials and Methods

### Ethics Statement

All experiments were carried out in accordance with the National Institutes of Health Guide for the Care and Use of Laboratory Animals (NIH Publication No. 80–23) and all procedures for this experiment were approved by the University Committee for the Care and Use of Animals at the University of Michigan (08497).

### Subjects

Forty-eight male Sprague-Dawley rats (Charles River Laboratories, Wilmington, MA) weighing 260 g at the time of arrival were housed individually in opaque single standard cages (27.7 cm X 20.3 cm) maintained on a LD 12∶12 cycle. Core body temperature was monitored in a subset of animals with intra-abdominal transmitters (pdt 4000 e-mitter-telemetry implants, Mini-mitter Inc., Bend, OR; *n* = 21), and all animals had running wheels for monitoring of activity (*n* = 48). **Figure S1** provides a detailed flow chart identifying the number of animals utilized throughout the study (number of animals that underwent each procedure) and the number of animals per condition that were included in the final analysis. All circadian data was analyzed off-line using Actiview software (Minimitter, Bend, OR).

### Experimental Timeline

Animals were acclimated to their home cages and LD cycle for a minimum of two weeks before undergoing surgery. All animals designated to undergo infusions of artificial cerebral spinal fluid (ACSF) or 192 IgG-saporin underwent surgery for telemetry implants or sham surgeries for this procedure, followed by two weeks for recovery, before being introduced to running wheels. Animals with SCN electrolytic ablation were allowed two weeks for acclimation to their environment before being introduced to running wheels. Animals had *ad libitum* access to food and water during the two-week baseline period. Afterwards, animals were mildly water deprived to ∼95% of their free feeding weight while having *ad libitum* access to food (Purina 5001; supplier: Frontier, Oxford, MI). Water deprivation occurred over seven days beginning with twelve-hour access on day one (starting 6 hours prior to their training time; ZT4 or ZT16, and continuing for 12 hours). Gradually over the next six days, animals were titrated down to a single one hour water access period from ZT4 to ZT 5 or ZT16 to ZT17, respectively. Following the two-week baseline period and gradual water deprivation, task-performing rats began operant training procedures to facilitate shaping on a task that measures sustained attention (SAT). Electrolytic lesion (EL) rats trained four hours after lights-on (ZT4), and the saporin-lesion (SL) and vehicle control (ACSF) rats trained at one of two times: four hours after lights-on (ZT4) or four hours after lights-off (ZT16). Locomotor activity during baseline and training phases was collected in 10-min bins using Vitalview software (Minimitter, Bend, OR). All task-performing animals were given free access to water for twenty minutes immediately following daily training in addition to the quantity of water (∼5 ml) obtained as reward during operant testing. Upon achieving a performance criteria (described below), rats were released into constant darkness (DD) for 14 days to assess strength of entrainment. During the initial 48 hours following release into DD, animals underwent complete water deprivation to eliminate time of water access as a potential cue. One ACSF infused animal training at ZT4 died before task acquisition was considered completed and was not included in performance, anticipation, or *free-run* analysis.

### Surgeries and Data Collection

Lesions using 192 IgG-saporin (Advanced Targeting Systems, San Diego, CA) were designed to selectively deafferent basal forebrain cholinergic projections to the SCN. Animals were anesthetized using isoflurane (2.5% iso, 95% oxygen) and placed in a stereotaxic instrument adjusted to level between bregma and lambda. Bilateralal injections were made using a 30 gauge needle attached to a 1 µL Hamilton syringe lowered once per hemisphere into the SCN. Rats received 0.4 µL per hemisphere of a 200 ng/µL of 192 IgG-saporin suspended in artificial cerebro-spinal fluid (ACSF). Control animals received surgery under the same protocol except that vehicle alone was infused (ACSF).

Animals with electrolytic ablation of the SCN underwent a single surgery when they reached 350–400 g to selectively ablate the SCN. A 50 µm stainless steel lesioning electrode (FHC; Bowdoin, ME) was lowered to four SCN locations (two/hemisphere). Complete details are available in the supplementary section (***Methods S1***). Electrolytic lesions were produced by passing DC current at 0.5 mA for 20 seconds. A total of 24 rats underwent electrolytic lesioning of the SCN and the 12 rats that showed complete arrhythmia based on running wheel activity, were selected for this study. Four animals from the SCN ablation group that began training were excluded at the conclusion of the study based on histological verification of damage to the optic chiasm or damage outside of the SCN.

### SAT Operant Methods

Operant training took place seven days per week. Behavioral training and testing was conducted in individual operant chambers (MedAssociates; St. Albans, VT) outfitted with two retractable levers, three red panel lights (2.8 W), and one red house light (2.8 W). Additionally, a water dispenser attached to a syringe pump, was located on the same wall as the panel lights and levers. Rats were removed from their home cages and placed in unlit chambers for five minutes prior to task onset. Operant chambers were housed within individual sound-attenuating cabinets. The shaping protocol for this task has been published in detail previously and is described in detail in Methods S1 [13]. Briefly, animals were trained to detect, discriminate, and respond to signal and non-signal events. Each correct response produced a water reward (30 µL for each hit or correct rejection) and incorrect responses (miss or false alarm) were not rewarded. Each operant session consisted of 162 trials. During the final stage of testing (referred to as the ‘SAT’), the house-light was illuminated increasing attentional demand. The addition of the illuminated house-light requires the animal to constrain their behavior toward observing the central panel during testing to optimize performance. Animals were required to maintain criterion performance (≥70% correct responses to the 500 ms signal trials, ≥70% correct responses to non-signal trials and fewer than that 20 omissions per session) for 3 consecutive sessions before task acquisition was considered complete.

### SAT Data Analysis and Statistics

Statistical analyses were carried out to determine differences in the number of days to criterion on the SAT (task acquisiton) and overall task performance at criterion. Days to criterion were tested using independent t-tests between lesion conditions within time of training or between time of training within a lesion condition. Mixed analyses tested the main effects and interactions of treatment conditions and signal duration. SAT performance yielded measures of hits (H), misses (M), false alarms (FA), correct rejections (CR), and omissions (O). Statistical analysis of hit percentage (%H), correct rejection percentage (%CR), and percent omissions (% O) was tested using independent t-tests using the criteria stated above. Comparison of group means between experimental groups and controls was performed using SPSS (SPSS Statistics Version 17.0; Chicago, IL).

### Circadian Analysis and Statistics

The phase onset (start) and offset (end) of activity relative to light-phase and SAT training were determined by the presence of 3 or more consecutive 10-min bins of activity (BOA) that were more than 10% of the daily mean activity and were analyzed for the last ten days of recorded activity following task acquisition and prior to release into constant conditions (dark:dark; DD). Activity ratios were determined using BOA counts (number of 10 min bins in which activity was present) during the light and dark period, rather than the total activity or peak of amplitude (since rats run most intensively during any light transition) to best assess the distribution of activity in relation to the light and dark-phase. LD ratios greater than 1.0 indicate a diurnal activity pattern whereas those less than 1.0 indicate a nocturnal activity pattern. Days of entrainment, determined by the number of days that showed activity at the time training would have occurred in LD (***figure S4***) or DD conditions, and overall LD ratio were compared between treatments using a one-way *ANOVA*. Significant main effects were further analyzed using *Tukey post-hoc* analysis. A probability value of *p*<0.05 was used as the criteria to determine statistical significance. Statistical analysis was performed using SYSTAT (Systat V13, Systat Software Inc., Chicago, IL).

## Results

### Quantification of Cholinergic Lesions

**Figure 1:**
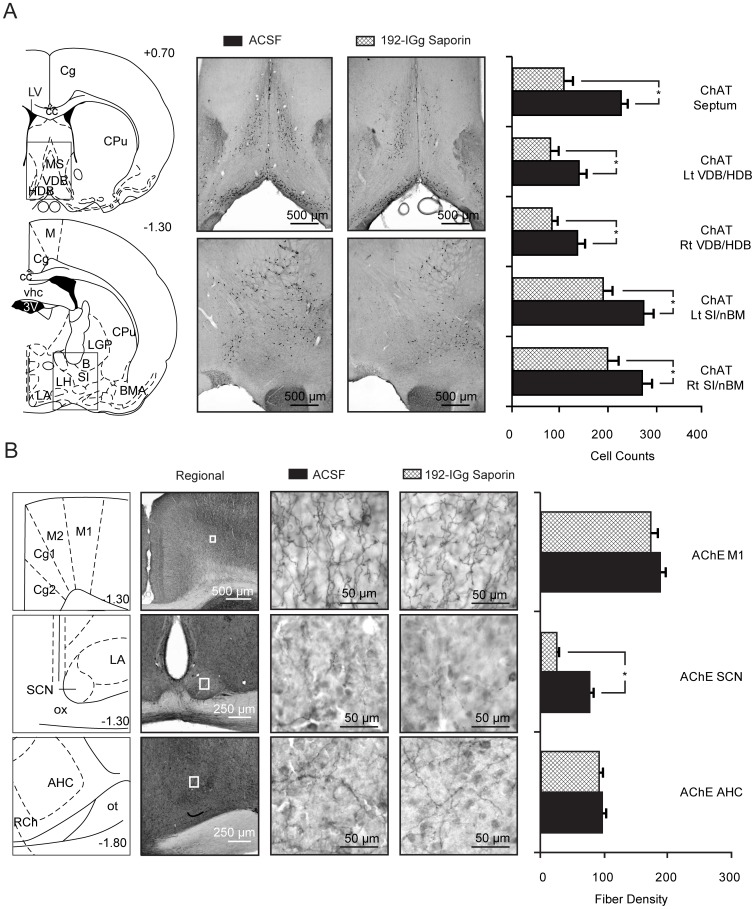
Anatomical results of neurotoxic lesions for animals used in this study. (Anterior-posterior levels based on Bregma are shown in schematics; scale for all photomicrographs is indicated in the lower right of image): *(* ***A*** *)* Left side provides schematic illustration of coronal sections shown in microphotographs on right. Box outline in schematic represents the boundary of the microphotographs for areas in which ChAT-positive cell bodies were counted. Images are representative for animals that received ACSF infusions (controls) or 192 IgG-saporin infusions. The bar graphs on the right indicate ChAT-positive cell counts for animals by group for each region quantified (* = *p*<0.05). *(* ***B*** *)* Left side represents schematic of regional sections shown in photomicrographs (center). Box outline in regional photomicrographs represent 40X images shown on right from ACSF and 192 IgG-saporin infused animals, respectively. Bar graph on the right indicate AChE+ fiber counts for animals by group for each region quantified (M1: layer 3/5, SCN, and AHC; * = *p*<0.01). Abbreviations: 3 V, third ventricle; AHC, anterior hypothalamus; B, basal nuc. of Meynert; BMA, basal medial amygdala; cc, corpus callosum; Cg, cingulate cortex; CPu, Caudate/Putamen; HDB horizontal diagonal band of Broca; LA, lateral anterior hypothalamus; LGP, lateral globus pallidus; LH, lateral hypothalamus; LV, lateral ventricle; M1, primary motor cortex; M2, secondary motor cortex; MS, medial septal nuc.; ot, optic tract; ox, optic chiasm; RCh, retrochiasmatic area; SCN, superchiasmatic nuc.; SI, substantia innominata; VDB, vertical diagonal band of Broca.

**Figure 1** provides histological results for animals with targeted infusions of 192 IgG-saporin into the SCN. We quantified the thoroughness of the saporin lesions using fiber density counts of acetylcholinesterase (AChE) in the SCN relative to control regions. We also addressed the regional specificity of SCN targeted infusion by quantifying changes in cholinergic expression in surrounding hypothalamic areas (anterior hypothalamus and the medial preoptic area) and motor cortex. Counts of AChE-positive fibers revealed a within-subjects effect of region (*F*(3,63) = 229.532, *p*<0.001) and a main effect of treatment group (*F*(1,21) = 15.602, *p*<0.001; ***figure 1B***). *Post-hoc* analysis found significant differences in fiber loss between saporin lesions and controls for the SCN (p<0.001), but not for motor cortex (p = 0.276), anterior hypothalamus (p<0.310), or the medial preoptic area (p<0.704). The loss of AChE+ fibers in the SCN was significant compared to the changes seen in adjacent regions of the hypothalamus and outlying cortical areas. The remaining cholinergic fiber staining in the SCN presumably originates from the brainstem cholinergic neurons including the pedunculopontine, laterodorsal tegmental and parabigeminal nuclei [18] which are spared by 192 IgG-saporin [27].

We also quantified how SCN localized infusions of saporin impacted the number of cholinergic cell bodies in the basal forebrain using choline acetyltransferase (ChAT) immunohistochemistry. We found both a within-subjects effect of region (*F*(4,84) = 34.851, *p*<0.001) and a main effect of treatment group (*F*(1,21) = 51.258, *p*<0.001; (***figure 1A***) suggesting the loss of cholinergic cell bodies in saporin infused animals were not localized to one particular region of the basal forebrain. *Post-hoc* analysis revealed significantly fewer ChAT+ cells for all regions quantified: septum (*p*<0.001); Left DB (*p* = 0.008); Right DB (*p* = 0.003); Left SI/nBM (*p* = 0.001); and the Right SI/nBM (*p* = 0.015) confirming that basal forebrain cholinergic innervation of the SCN arises from a distributed network of cells in disparate locations as previously described [18], [28].

### Influence of Cholinergic Lesions on Measurements of Daily Activity and Body Temperature

**Figure 2:**
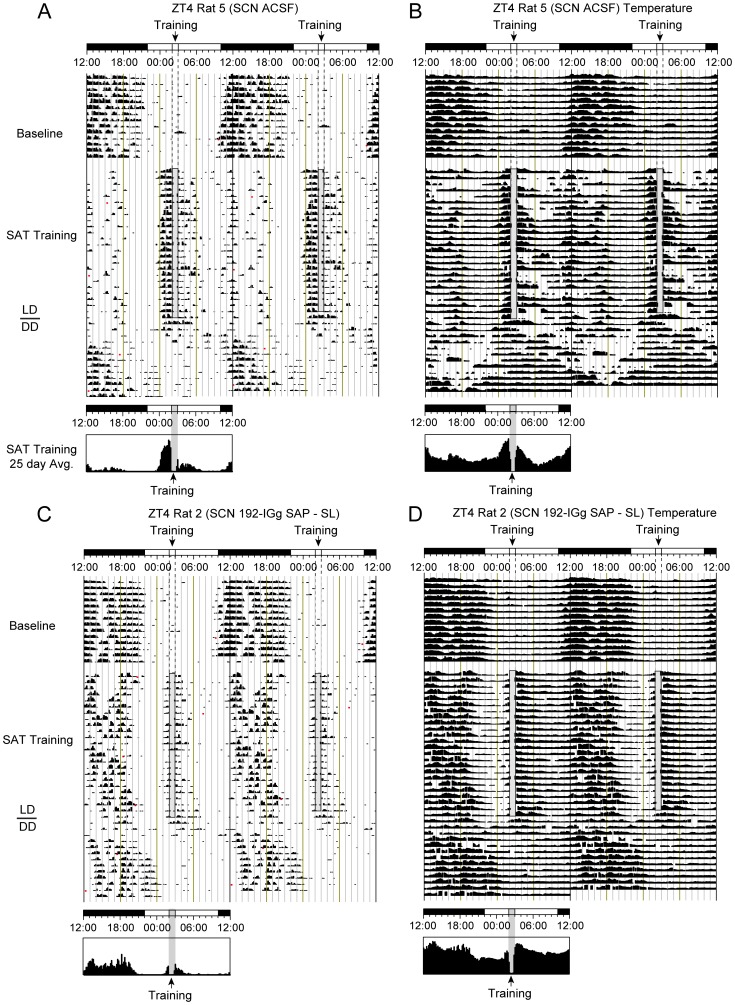
Representative double-plotted running wheel and temperature records for animals training on the Sustained Attention Task (SAT) at ZT4 following ACSF (A&B) or 192 IgG-saporin infusions (C&D) into the SCN. Each line represents 48 hours. SAT training (ZT4) is relative to the topmost LD bar (where dark bar = lights-off). The shaded column represents the task training period where daily water is available including the approximate 40 min SAT session in which animals are absent from their home cages and the 20 minute free water period that follows training. The last day of training corresponded to the final day of LD conditions. Actograms are separated into 2 phases: The first phase represents 14 days of baseline activity prior to SAT training but following ACSF or saporin infusions into the SCN. The second portion of the actogram illustrates the final 25 days of SAT training and the subsequent 14 days of constant conditions (DD). Time histograms below provide 24-hour averages of running wheel activity (**A&C**) or body temperature (**B&D**) rhythms for the final 25 days of training shown above. Shaded columns represent time of training. *(* ***A*** *)* Activity and *(* ***B*** *)* temperature record of ZT4 animal with ACSF (control) infusions into the SCN. Note that animal shows a substantial daily anticipation for time of daily training and a primarily diurnal activity pattern. *(* ***C*** *)* Activity and *(* ***D*** *)* temperature record for ZT4 animal with 192 IgG-saporin infusions into the SCN. Daily anticipation is substantially reduced and this animal maintains a primarily nocturnal phenotype. Free-run activity in the lesioned animal, relative to the time training would have occurred, is robustly shorter compared to the control animal.

**Figure 3:**
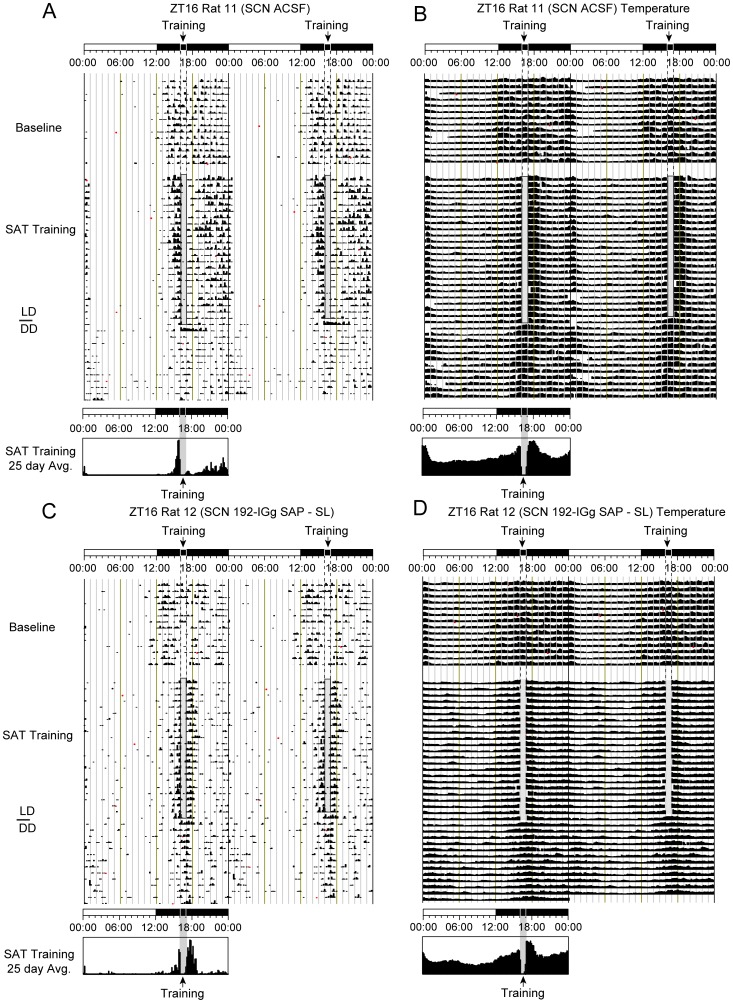
Representative 48 hour double-plotted running wheel actogram and temperature record for ACSF (A&B) or 192 IgG-saporin infused animals (C&D) training on the SAT at ZT16. SAT training is relative to the topmost LD bar (where dark bar = lights-off). The shaded column represents the task training period where daily water is available including the approximate 40 min SAT session in which animals are absent from their home cages and the 20 minute free water period that follows training. The last day of training corresponded to the final day of LD conditions. The first 14 days represent baseline activity of animals with ACSF or saporin infusions into the SCN prior to SAT training. The second portion of the actogram illustrates the final 25 days of training of SAT training and the subsequent period of constant conditions (DD). Time histograms below provide averages of running wheel activity for the final 25 days of training shown above on a 24-hour scale. Shaded column represents time of training. *(* ***A*** *)* Double-plotted actogram and *(* ***B*** *)* temperature record of ZT16 animal with ACSF infusions. Note that animal shows a robust increase in activity prior to daily training. *(* ***C*** *)* Actogram and *(* ***D*** *)* temperature record of ZT16 animal with 192 IgG-saporin infusions into the SCN. Daily anticipation for task training is reduced relative to control animals with the greatest activity coinciding with the period after the animal returns from training.

We next compared the effects of localized 192 IgG-saporin (SL) infusions into the SCN on the activity of nocturnal rodents training within or outside their endogenous active phase. Because body temperature is considered a more reliable marker of SCN output than activity [29], we also measured how cholinergic deafferentation of the SCN affected body temperature rhythms. Initial comparisons revealed that saporin infusions had no discernible influence on activity or temperature rhythms of animals housed in a 12∶12 LD cycle under baseline non-performing conditions. Both artificial cerebral spinal fluid (ACSF) controls and SL animals showed robust synchronization to the LD cycle following surgery and prior to the initiation of daily training that did not differ from their pre-surgery baseline (activity: *F*(1,46) = 0.085, *p* = 0.772; temperature *F*(1,40) = 0.180, *p* = 0.673) or from one another (activity: *F*(3,20) = 1.09, *p* = 0.375; temperature: *F*(3,17) = 0.463, *p* = 0.712; ***figure 2A–2D*** ***, and*** ***figure 3A–3D***; *baseline period*). This result indicates that elimination of the basal forebrain cholinergic input to the SCN did not impair the establishment or continuous expression of photic-mediated entrainment in agreement with other studies [28], [30].

Animals were randomly divided into 2 groups: those that would be trained four hours after lights-on (ZT4) or four hours after lights-off (ZT16). Representative running wheel records from task performing animals with SL of the SCN or corresponding ACSF controls are shown in **figure 2A** **and** **figure 2C** respectively (ZT4); and **figure 3A** **and** **figure 3C** respectively (ZT16). Temperature records from the same animals, over the same period as the adjacent activity records, are shown in **figure 2B** **,** **figure 2D**, **figure 3B** **and** **figure 3D**.

### Cholinergic Lesions of the SCN Reduce Activity and Core Temperature during the Light-phase in ZT4 Trained Animals

**Figure 4:**
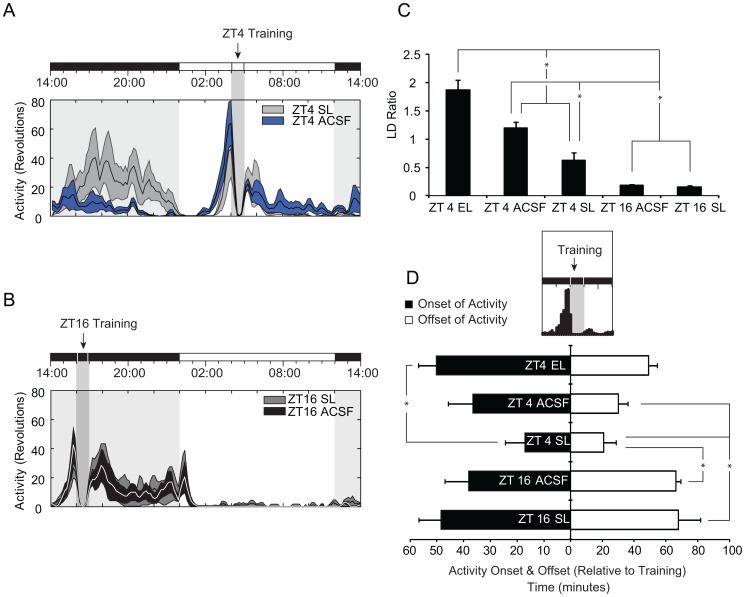
Mean activity and phase synchrony for SAT trained animals by treatment group. *(* ***A*** *)* 24 hour binned average of ZT4 trained animals. SAT training (ZT4) is relative to the topmost LD bar (where dark bar = lights-off). The shaded column represents the task training period where daily water is available including the approximate 40 min SAT session in which animals are absent from their home cages and the 20 minute free water period that follows training. The X-axis is plotted in zeitgeber time (ZT) and y-axis represents wheel revolutions with activity grouped into 10 min bins. Mean histograms are taken from the last 10 days of SAT training before entering constant conditions. ZT4 IgG-saporin lesioned - SL (light gray), and ZT4 ACSF controls (blue) mean averages are plotted ± SEM (shading). *(* ***B*** *)* 24 hour binned average of ZT16 trained animals. Training is relative to the topmost LD bar and the shaded column represents the SAT training period in which animals are absent from their home cages. ZT16 SL (dark gray), and ZT16 controls (black) mean averages are plotted ± SEM (shading). (***C*** *)* Mean Light-Dark (LD) activity ratios from final 10 days of training on the SAT ± SEM (* = *p*<0.05). LD ratio is calculated by taking the number of 10-min bins of activity during the light-phase and dividing by the number of 10-min bins of activity during the dark-phase with LD activity ratios less than 1.0 indicative of nocturnal behavior. ZT4 electrolytic-lesioned (EL) animals show an LD ratio significantly higher than all other treatments. ZT4 controls also differed significantly from ZT4 SL animals and animals that trained at ZT16. *(* ***D*** *)* Mean phase relationship between onset and offset of locomotor activity and time of sustained attention task (SAT) training. Activity onset and offset in minutes relative to training is plotted for each condition (minutes ± SEM; * = *p*<0.05).

We had previously demonstrated that timed daily performance during the light-phase on a task of sustained attention (**SAT**) in nocturnal rodents results in animals with a diurnal phenotype and that the level of diurnality was highly correlated with the cognitive demands of the task. [12]–[13]. Comparisons of activity and temperature records from ZT4 trained SAT animals receiving bilateral infusions of ACSF (***figure 2A*** ***and*** ***figure 4A***) did not differ from those previously published; however, activity and temperature records of SCN-saporin infused animals (***figure 2C*** ***and*** ***figure 4A***) were noticeably different from those of ACSF infused controls.

In order to quantify these differences, we assessed activity and temperature with three approaches: we first compared daytime activity or diurnality by looking at the ratio of light-activity to dark-activity. The LD ratio represents the average activity in the light-phase divided by the average activity in the dark-phase, with a value less than 1.0 representing a nocturnal phenotype and a value greater than 1.0 representing a diurnal phenotype. Second, we quantified differences in activity or temperature anticipation relative to the onset and offset of task training time, and third, we measured entrainment by quantifying the duration of activity or temperature changes relative to the time training would have occurred under conditions where all environmental cues had been removed (constant conditions).

ZT4 trained ACSF infused animals show enhanced activity during the light-phase and greater diurnality (1.20±0.11) compared to all other training groups (*ANOVA* (*F*(4,26) = 37.849, *p*<0.001; ***figure 4C***). ZT4 controls were significantly more diurnal than dark-phase training groups (LD = 0.18±0.02; ZT16 control, *p<*0.001; ZT16 SL, *p*<0.001) or ZT4 saporin lesioned animals (LD = 0.63±0.13; *p* = 0.041). ZT4 SL animals showed relatively minimal daytime activity during SAT training, remaining predominantly night active, and not differing statistically from either dark-phase training group (ZT16 SL, *p* = 0.076; or ZT16 controls, *p* = 0.160). As expected, all ZT16 control and ZT16 SL animals had LD ratios of less than 1.0, indicative of nocturnal behavior.

**Figure 5:**
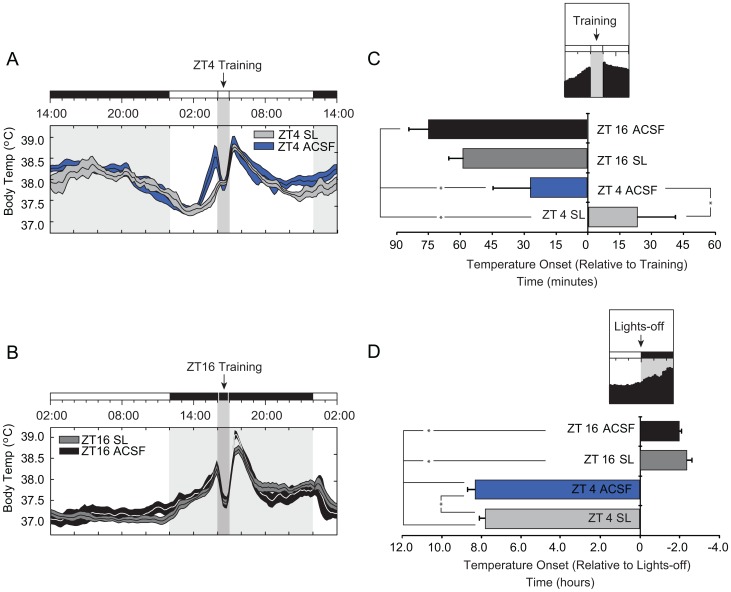
Mean temperature records and phase synchrony for SAT trained animals by treatment group. *(* ***A*** *)* 24 hour binned average of ZT4 trained animals. SAT training (ZT4) is relative to the topmost LD bar (where dark bar = lights-off). The shaded column represents the task training period where daily water is available including the approximate 40 min SAT session in which animals are absent from their home cages and the 20 minute free water period that follows training. Values of zero - occurring when animals are removed from their home cages for training (ZT4-ZT5, or ZT16-ZT17 for ZT4 and ZT16 trained animals, respectively), were adjusted to reflect daily mean values. X-axis is plotted in zeitgeber time (ZT) and y-axis represents mean body temperature grouped by 10 min bins. Histograms are taken from the last 10 days of SAT training, before entering constant conditions. ZT4 SL (light gray), and ZT4 controls (blue) mean averages are plotted ± SEM (shading). *(* ***B*** *)* 24 hour binned average of ZT16 trained animals. ZT16 SL (dark gray), and ZT16 control (black) mean averages are plotted ± SEM (shading). (***C*** *)* Mean phase relationship between rises in body temperature (crossing above daily mean) relative to time of SAT training. Activity onset in minutes relative to training time is plotted for each condition (minutes ± SEM; * = *p*<0.05). Note all animals show significant daily increase before task training with the exception of ZT4 SL animals. (***D*** *)* Mean phase relationship between rises in body temperature (crossing above daily mean) relative to time of lights-off. Activity onset in minutes relative to dark-phase is plotted for each condition (minutes ± SEM; * = *p*<0.05).

As body temperature rhythms are a direct indicator of SCN output [29], we examined whether saporin lesions influenced daily temperature rhythms in a manner similar to those measured for activity rhythms. We compared mean temperatures and temperature distributions across both the light-phase and the dark-phase of the LD cycle (***figure 5A*** **and** ***figure 5B***). Temperature data were collected from a subset of all animals (21 of 24: ZT4 controls: n = 5; ZT4 SL: n = 6; ZT16 controls: n = 5; ZT16 SL: n = 5). Repeated measures analysis revealed an effect of time on body temperature across both the lights-on phase (*F*(71,1207) = 16.395, *p*<0.001), and lights-off phase (*F*(71,1207) = 19.097, *p*<0.001) confirming a cyclic pattern of temperature regulation across the LD cycle in all treatment groups. ZT4 animals, however, demonstrated a bimodal temperature rhythm with each animal showing a peak corresponding to their training time (ZT4) and a second peak during the dark-phase (*F*(3,17) = 35.736, *p*<0.001; ***figure 5A***). While the bimodal peak was conserved in ZT4 SL animals, the amplitude and duration of light-phase activity were substantially attenuated by saporin lesions (ZT4 and ZT4 SL, *p* = 0.044). In contrast, ZT16 trained animals show a unimodal peak centered in the middle of the dark-phase in both ACSF and SL conditions.

### Cholinergic Lesions of the SCN Block Anticipatory Increases in Activity and Temperature in ZT4 Trained Animals

Measures of overall light-phase or dark-phase activity provide insight into how cognition modifies circadian output; however, the strength of that association can be more specifically quantified by amplitude and duration of daily activity that occurs in anticipation of the training time and the extent to which activity persists after training. Therefore, we measured anticipation for daily task training across two measures: the onset of activity relative to time of task (*F*(4,26) = 2.90, *p* = 0.041; ***figure 4D:*** ***black bar***) and the activity offset relative to the time of task (*F*(4,26) = 5.50, *p* = 0.002; ***figure 4D:*** ***white bar***). *Post-hoc* analysis revealed that anticipatory increases in activity (in minutes) occurs earlier for ZT4 controls (36.6±9.1) relative to ZT4 SL animals (17.1±7.2; *see* ***figure 4D***) suggesting cholinergic lesions reduce the strength of anticipation for the time training will occur in light-phase trained animals.

We also quantified synchronization to daily task training by measuring the anticipatory rise in body-temperature, relative to the daily-mean, for task onset (*F*(3,17) = 2.468, *p* = 0.002; ***figure 5C***). ZT4 control animals showed significant earlier onset of temperature increase relative to task onset compared with ZT4 SL animals (***figure 5A*** ***and*** ***figure 5C***, *p* = 0.026). Both ZT16 groups showed substantially earlier elevation than both daytime training groups (ZT4 control, *p* = 0.039; ZT4 SL, *p*<0.001), however, because this increase is coincident with the onset of the dark-phase, it is difficult to determine what portion of the rise is specifically attributable to task anticipation (***figure 5B***).

In addition to anticipatory rises in temperature relative to the time-of-training, body temperature differed in anticipation of the onset of the dark-phase across treatment groups (*F*(3,17) = 6.841, *p*<0.001; ***figure 5D***). For dark-phase trained groups, the increase above the daily mean occurs at or about the dark-phase transition, with ZT16 controls showing a slightly earlier rises in temperature relative to SL animals (***figure 5B***, ***figure 5D***). ZT4 controls showed significantly earlier rises in body temperature compared to all other training groups (ZT16 controls, *p* = 0.005; ZT16 SL, *p*<0.001; ZT4 SL, *p*<0.015). This finding suggests light-phase trained control animals express a bimodal distribution of daily temperature rhythms that does not occur in ZT16 trained and is more robust than the distribution seen in ZT4 saporin lesioned animals.

### Cholinergic Lesions of the SCN Reduced Days of Free-run Under Constant Conditions

Finally, we measured the strength of entrainment based on number of days that activity or temperature changes persisted under conditions of total darkness (DD). If periodic changes associated with daily training continue in the absence of all external cues, including task cues or the light-dark cycle (*free-run*), the physiology or behavior that is synchronized by the task provide evidence of entrainment. In order to quantify entrainment, animals on the final day of training under LD conditions were returned to their cages and watered as normal. In the case of ZT4 animals, the onset of the dark-phase at ZT12 began the 14 day DD condition. ZT16 animals were returned to the dark-phase where lights remained off until the end of the DD period. All animals were left undisturbed except for minimal random checks on food, water, and health under red light conditions. The number of days of activity at the time training would have occurred under *free-run* conditions were greatest for animals training at ZT16 (SL = 13.9±0.1 days; control = 13.8±0.2 days). All ZT16 averages were longer than those seen in ZT4 trained animals (ZT4 control = 3.6±1.0 days; ZT4 SL = 2.5±0.3 days; *p*<0.01). Activity for the DD condition is shown in the last 10–14 lines of individual actograms for each treatment condition in **figure 2** **and** **figure 3**. Note the final day of training coincides with animals entering the DD condition as demonstrated by the gray bar.

Days of temperature entrainment under *free-run* conditions also persisted for more days in animals training at ZT16 (SL = 13.3±0.3 days; controls = 13.7±0.2 days). ZT16 averages were greater than ZT4 averages (ZT4 controls = 8.3±1.4 days; ZT4 SL = 1.8±0.3 days; *p*<0.001). ZT4 control animals also showed more robust entrainment as measured by continuous days of daily rises in body temperature compared to ZT4 SL animals. Temperature records for the *free-run* period are shown in the bottom 10–14 lines of **figures 2B & 2D** **and** **figures 3B & 3D**.

Based on our early assessment, ZT4 ACSF animals showed a more pronounced incoherence between activity and temperature rhythms that was not apparent in other treatment groups that maintained strong activity/core temperature synchrony. This is most noticeable in comparisons between activity and temperature records coincident with the onset of DD (*i.e.,* ***figure 2A*** ***and*** ***figure 2B***). The differences in the origin and intensity of the activity and temperature rhythms during the *free-run* period illustrates the conflict in underlying oscillators that may model the internal desynchrony (ID) noted in human chronic shift-workers [31]–[33].

Across all metrics measured we found significant differences between animals training at ZT16 and ZT4, irrespective of lesion condition. In addition, results from the saporin lesioned ZT4 animals indicate that basal forebrain cholinergic input to the SCN plays an essential role in promoting cognitive activity as cholinergic depletion of the SCN significantly attenuates task-mediated markers of synchronization and entrainment (*i.e.,* anticipation to activity onset, LD ratio, and *free-run* under constant conditions; ***figure 2*** ***,*** ***figure 3*** ***,*** ***figure 4*** ***, and*** ***figure 5***). These findings also reveal that cholinergic signaling in the SCN may convey timing information for salient events, potentially serving as an SCN timing signal [34]–[35].

### Cognitive Training of Nocturnal Rodents during the Light-phase Promotes Internal Desynchrony

As described above, temperature rhythms and activity rhythms in task-performing animals do not always share the same phase relationship. In particular, it appears that daytime-trained animals have a higher level of desynchrony based on comparisons of body temperature and activity rhythms. In order to quantify this, we compared individual body temperature and activity rhythms for the 21 animals that had measures of both across the light-cycle and dark-cycle. Measurements were made relative to their baseline or non-performing condition as a measure of desynchrony (***figure S2***). Detailed analysis (see ***results S1***) revealed that control animals training at ZT4 had the most profound level of ID of any of the training groups and that cognitive activity at a time outside of the endogenous activity period produced a state of desynchrony that was not seen in animals trained during the dark-phase.

### Is the SCN Necessary for Cognitive Entrainment?

**Figure 6:**
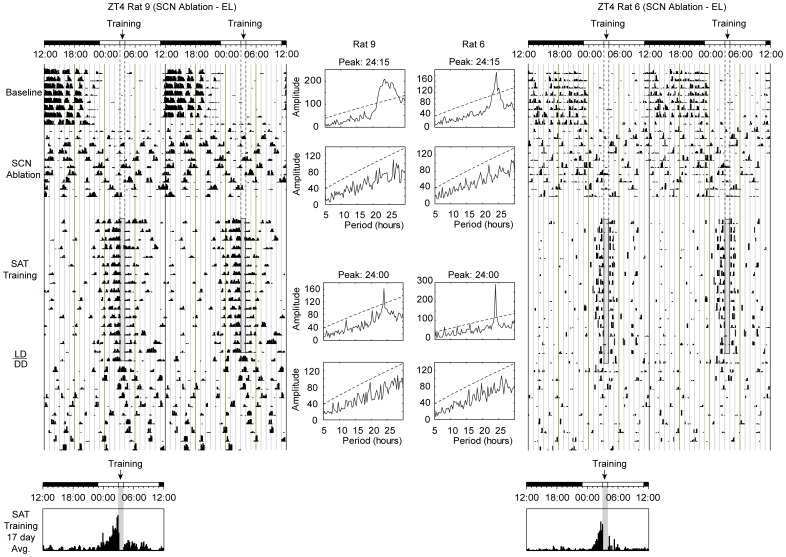
Double-plotted actograms for two SCN ablated (electrolytic lesion - EL) animals training on the SAT at ZT4. Each line represents 48 hours of locomotor activity collected via running wheels. SAT training (ZT4) is relative to the topmost LD bar (where dark bar = lights-off). The shaded column represents the task training period where daily water is available including the approximate 40 min SAT session in which animals are absent from their home cages and the 20 minute free water period that follows training. The last day of training corresponded to the final day of LD conditions. Circadian actograms are separated into 2 phases with 4 observable activity states: The first phase represents the final 8 days of baseline activity prior to surgery and lesioning of the SCN, followed by the first 10 days of post-lesion activity prior to the onset of SAT training. The second portion of the actogram illustrates the final 17 days of SAT training and the subsequent 10 days of constant conditions (DD). Time histograms below provide averages of running wheel activity for the final 17 days of training shown above on a 24-hour scale. Shaded column represents time of training relative to the LD cycle. In both cases animals show an arrhythmic phenotype following lesioning of the SCN in both LD and DD environments. *(Center)* Chi-square (χ^2^) period analysis from 10 consecutive days of activity are represented for pre-surgery (*top*), post SCN-lesioning (*middle-top*), SAT training (*middle-bottom*), and post-training period (*bottom*) for the corresponding actograms. Values above the dashed line represent significant periodicities *(p = 0.05)* with peak activity periods noted in instances of significance.

In addition to testing how the loss of basal forebrain cholinergic inputs to the SCN impact SAT entrainment, we tested the necessity of the SCN for cognitive entrainment by training animals with electrolytic lesions (EL) of the SCN. For a variety of stimuli, including food and methamphetamine, entrainment can occur in the absence of the SCN [24]–[26]. We tested whether cognitive activity could similarly produce anticipation for daily training and continued activity following removal from task in SCN-lesioned animals. **Figure 6** shows circadian activity from two animals’ representative of the eight EL animals that met inclusion criteria for this study.

Bilateral SCN ablation resulted in a pronounced and persistent arrhythmia in both the presence and absence of a light-dark cycle (***figure 6***; *SCN ablation period*). SAT training, however, restored daily periodicity in activity rhythms at 24-hour intervals and a produced a robust increase in activity anticipating and following the time of daily training. Animals with SCN ablation also had the highest LD ratios (1.87±0.18) and strongest anticipation for the SAT when compared to other light-phase (ZT4) training groups (*F*(3,17) = 2.468, *p* = 0.002; *see* ***figure 4D***; *p* = 0.034; ***figure S3A***). Despite showing enhanced light-phase activity and earlier anticipation relative to task training, ZT4 EL animals showed a modest amount of *free-run* activity (2.9±0.8 days) following cessation of training (150+ continuous days) before returning to an arrhythmic condition. This finding suggests that oscillators entrained by attentional performance are unable to sustain synchronized activity for more than a few days without periodic signals analogous to those noted for other non-SCN oscillators [9], [25]. Additionally, the observation that ZT4 EL animals training on the SAT show unparalleled light-phase activity and anticipation of training time is also consistent with the theory that cognitive activity entrains oscillator(s) outside of the SCN and is capable of organizing circadian activity independently.

### Ablation of the SCN Impairs SAT Task Acquisition

Finally, we examined how SCN ablation influences task acquisition and performance in SAT trained animals. Because lesions of the SCN have been shown to impair novel object recognition [36] and the attenuation of circadian rhythms is correlated with impaired spatial learning in some tasks [37], we analyzed whether SCN lesions impaired learning of the sustained attention task. Because unintended damage to the optic chiasm could result in deficits in task performance, subjects with visible damage to the optic structures were excluded from analysis. ZT16 animals acquired the task faster than ZT4 trained animals (*F*(1,29) = 4.456, *p = *0.044) as has been described previously [12]. SL and control animals trained at ZT4 did not differ significantly (*p* = 0.384) nor did ZT16 SL and ZT16 control animals (*p* = 0.854). However, statistical analysis revealed significant differences between ZT4 EL and the ZT4 control animals (*t*(11) = 2.251, *p* = 0.049) confirming that a functional SCN facilitates task acquisition.

## Discussion

The established relationship between attentional demand, cortical acetylcholine (ACh) release, and the anatomical connectivity between cholinergic regions and the SCN led us to hypothesize that the changes in circadian entrainment associated with cognitive training are mediated through mechanisms of cholinergic signaling. Furthermore, we speculated that cholinergic signaling in the SCN presumably originates from corticopetal cholinergic neurons in the basal forebrain which are recruited during attentional performance. In order to test this hypothesis, we selectively removed forebrain cholinergic projections to the SCN with targeted infusions of the neurotoxin 192 IgG-saporin. Our results reveal that deafferentation of forebrain cholinergic inputs to the SCN attenuates diurnal activity (*i.e.,* anticipation to onset of training time and LD ratio) that ensues from SAT training during the light-phase (***figure 2A*** ***,*** ***figure 2C*** ***,*** ***figure 4A*** ***,*** ***figure 4C*** ***, and*** ***figure 4D***). Animals with saporin-lesions of the SCN also had reduced task entrainment as measured by the number of days of continuous activity, relative to the time training would have occurred DD conditions. ZT4 trained controls showed more days of *free-run* activity (3.6±1.0 days) and continuous rises in temperature (8.3±1.4 days) relative to ZT4 SL animals (activity, 2.5±0.3 days; temperature, 1.8±0.3 days). The anatomical specificity of the 192 IgG-saporin-lesions (***figure 1***) combined with the behavioral data provide support for the hypothesis that basal forebrain cholinergic signaling to the SCN promotes light-phase activity during cognitive training.

The effects of SCN ablation also revealed that cognitive performance is capable of organizing daily rhythms independent of the SCN and is consistent with the hypothesis that oscillator(s) entrained by cognitive activity exist outside of the SCN. The synchrony of SCN-ablated animals was more robust, as measured by duration (minutes) and amplitude (revolutions/minute) of anticipatory activity to the time of task onset and overall LD ratios than all other daytime training groups. These findings parallel those described for other SCN independent non-photic entrainable oscillators including those entrained by food or methamphetamine, in that SCN ablation is accompanied by enhanced anticipatory activity for non-photic cues [10], [24]–[26]. If entrainment occurs in the absence of the SCN, the behavior is necessarily under the control of non-SCN oscillators which take on a more prominent role in modulating circadian output when uncoupled from the SCN. Importantly, our results reveal that non-SCN cognitive oscillators were not well synchronized in the absence of sustained cognitive performance (*i.e.,* ZT4 EL animals had arrhythmic wheel activity prior to SAT training, but eventually became synchronized to daily task performance, including a highly anticipatory diurnal rhythm, which quickly disappeared following the cessation of training and a return to arrhythmia within several days; *see* ***figure 6***). This rapid return to desynchronized activity suggests that cognitive oscillators are unable to maintain synchrony without external periodic cues (*i.e.,* SAT).

Results from animals with SCN ablations also support the hypothesis that the SCN is important for task acquisition and performance. Lesions of the SCN have profound effects on novel object recognition in hamsters [36] and age related dampening of circadian rhythms impairs development of conditioned place preference [37]. Acquisition was compromised in ZT4 EL animals with time to criterion averaging 35 percent longer relative to corresponding controls. Furthermore, ZT4 EL animals took substantially longer to synchronize to daily SAT training (*see* ***figure S3B***). Importantly, there were no significant differences across treatment groups for omitted trials during performance. As the number of omissions directly reflects a measure of water satiety or effort, this suggests the motivation to perform did not differ between time-of-day or lesion condition in a way that could explain this difference. It is also worth noting that since all ZT4 EL animals eventually reach criterion performance (*see methods*) it is unlikely that a high level of performance would be possible if damage to areas important for visual discrimination and cue detection had occurred. ZT4 EL animals additionally demonstrate a masking effect around each change in light-phase, which was not reported in other studies where lesions of the SCN extended into the optic chiasm [25]. Given that the SCN serves to synchronize various non-SCN oscillators; it is possible that in the absence of an SCN, oscillators sensitive to daily cognitive training take much longer to become synchronized to the signal and each other.

This interpretational framework allows us to speculate where oscillators associated with cognitive entrainment might exist and through what means they become entrained. It is possible that timed daily performance and associated cholinergic increases could be acting to modulate previously described oscillators (*e.g.,* water or food) making their impact more influential on behavior than has been previously reported [38]–[40]. Water has been described as a weak zeitgeber that produces no measurable anticipation in running wheel monitored rats when access is limited to the light-phase [40]. Although, water has been shown to produce entrainment in some SCN ablated hamsters, it only appears to do so if food intake (>50%) is largely limited to the period where water is available [39]. In a recent study we reported that SAT performing rats show modest intake of food during the 4 hour period following task training that represents 15–20% of total daily intake [41]. If large caloric intake is an important part of daily food entrainment, it seems unlikely that food entrainment plays a substantial role in mechanisms of cognitive entrainment. Further studies will be necessary, however, to determine if task evoked cholinergic neurotransmission influences the food-entrainable oscillator (FEO) in a way that promotes cognitive entrainment.

In contrast, it is also plausible that timed cholinergic signaling associated with task-performance could provide an entraining signal to novel unidentified oscillators. A number of potential cholinergic clock gene expressing targets are influenced by daily SAT performance and therefore could serve as candidate oscillators (e.g., prefrontal cortex, hippocampus [42]–[44]). Future experiments are necessary to identify candidate oscillators as well as identify the mechanisms by which they become synchronized.

**Figure 7:**
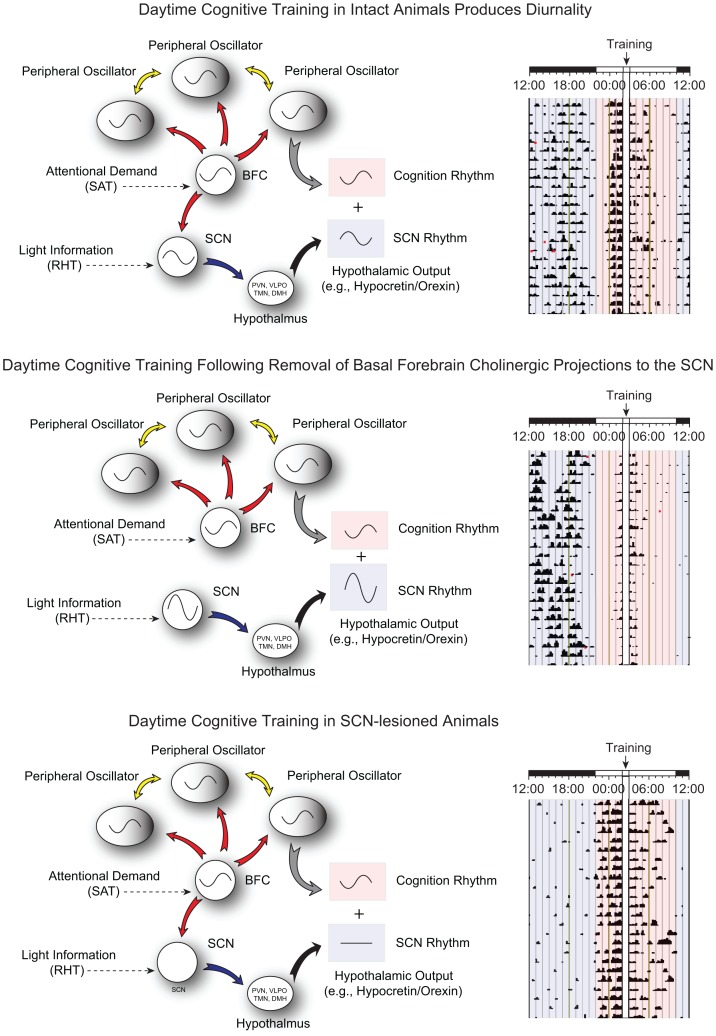
Proposed model of cognition mediated cholinergic interactions on circadian rhythms. *(Top)* Cholinergic signaling has the potential to influence oscillators of the SCN or peripheral oscillators of the CNS. Feedback of peripheral oscillators in turn, influences circadian rhythms by modulating SCN hypothalamic output directly or through indirect mechanisms. SCN output is relayed through nuclei of the hypothalamus thereby synchronizing non-SCN oscillators that regulate physiology and activity. Cognitive performance during normally inactive periods entrains peripheral oscillators to a phase *opposite* of those entrained by photic input to the SCN. Cholinergic input to the SCN attenuates rhythms generated by photic entrainment resulting in conflicting rhythms influencing activity. Animals trained under this conditions show markers of photic entrainment and cognitive entrainment resulting in an internally desynchronized state where animals are neither entirely diurnal nor nocturnal (top-right). *(Middle)* Removal of cholinergic projections to the SCN largely attenuates light-phase activity promoted by cognitive training and results in activity that is driven primarily by photic cues originating from the retina and terminating in the SCN (middle-right). Some level of daytime activity remains but it is largely overridden by SCN mediated light cycle entrainment. *(Bottom)* SCN ablated animals show a strong diurnal phenotype. In the absence of the SCN, animals show no synchrony to the light-dark cycle allowing non-photic (peripheral) oscillators to drive behavioral activity unobstructed (bottom-right). Abbreviations: BFC, basal forebrain cholinergic; DMH, dorsal medial hypothalamus; PVN, paraventricular nucleus; RHT, retinohypothalamic tract; SAT, sustained attention task; SCN, suprachiasmatic nucleus; TMN, tuberomamillary nucleus; VLPO, ventrolateral preoptic nucleus.

**Figure 7** provides a hypothetical summary of how daily performance could result in entrainment to daily cognitive activity. The data support a model where task related cholinergic signaling influences oscillators, both at the SCN, and at unidentified regions recruited during task performance. Over time, as in the case of SCN ablated animals, unidentified oscillators come to express a coherent state of synchrony that can be measured by biological or physiological phase markers. Animals trained during the dark-phase (ZT16) share a common phase of synchrony as non-SCN oscillators synchronize to task performance and light-driven oscillators of the SCN promote activity at the same time of day. ZT4 training results in a condition where cognition-induced oscillators compete against light mediated activity rhythms to influence behavior. This interaction is analogous to those proposed to mediate food anticipatory activity (FAA) that requires attenuation of SCN rhythms for the expression of FAA during the light-phase [9]–[10]. The exact mechanism by which cholinergic signaling could suppresses photic-driven SCN rhythms is unknown; however, cholinergic agonists do advance the timing at which peak firing rates occurs in SCN slices [21] and salient stimuli promote changes in SCN peptide output that are out of phase with light-mediated SCN peptide output [35], [45]. Future studies are necessary to determine the role of cholinergic signaling in promoting light-phase activity through SCN interactions.

Collectively, our results support a model of competitive interactions between non-SCN brain oscillators and the photic-driven SCN in rodents trained on a cognitively demanding task during the day. While non-SCN oscillators can entrain to a multitude of external cues, entrainment by cognitive activity represents a unique condition: it is internally motivated and the behavioral effect is not modulated by externally presented stimuli. Research on interactions between the basal forebrain cholinergic systems and circadian rhythms offer particular relevance to the change in SCN activity seen with old age: consequences of which include severe sleep disturbances, decreased sensitivity to environmental cues, a slowed SCN resynchronization speed, and weakened internal coupling of peripheral oscillators [46]. Positive correlations have been found between cholinergic cell-death in the basal forebrain and impaired cognition in older individuals with dementia and Alzheimer’s disease [47]. Recent studies suggest that some of these age-related effects on the SCN may be stimuli specific [48], and the close relationship between circadian activity and cholinergic mediated entrainment suggested by these results offers the potential of prospective therapies for circadian disruptions.

Finally it is important to note that cognitive training during the light-phase in normally nocturnal rats resulted in a severe state of internal desynchrony. This was evident by the asynchronous phase relationships between core body temperatures and motor activity and is consistent with the ID noted in chronic shift-workers [31]–[33]. Internal desynchrony was not seen in animals trained during the dark-phase and suggests that future research with this model could offer the potential to explore the long-term risks of circadian disorganization.

## Supporting Information

Figure S1 Study design and flowchart of animals used in analysis.
(TIF)

Figure S2 Comparisons of internal desynchrony (ID) during task training.
(TIF)

Figure S3 Entrainment rate during task acquisition and mean daily rhythms of SCN ablated animals.
(TIF)

Figure S4 Activity records of animals removed from task in LD conditions.
(TIF)

Methods S1 (DOCX)

Results S1 (DOCX)

## References

[1] Moore RY (1983) Organization and function of a central nervous system circadian oscillator: the suprachiasmatic hypothalamic nucleus. Federation Proceedings 42: 2783–2789. 6135628

[2] Stephan FK, Zucker I (1972) Circadian-Rhythms in Drinking Behavior and Locomotor Activity of Rats Are Eliminated by Hypothalamic-Lesions. Proceedings of the National Academy of Sciences of the United States of America 69: 1583–&. 10.1073/pnas.69.6.1583 PMC426753 4556464

[3] Maywood ES, Mrosovsky N, Field MD, Hastings MH (1999) Rapid down-regulation of mammalian period genes during behavioral resetting of the circadian clock. Proc Natl Acad Sci U S A 96: 15211–15216. 10611364 10.1073/pnas.96.26.15211 PMC24799

[4] Mistlberger RE, Skene DJ (2004) Social influences on mammalian circadian rhythms: animal and human studies. Biol Rev Camb Philos Soc 79: 533–556. 15366762 10.1017/s1464793103006353

[5] Cain SW, Ralph MR (2009) Circadian modulation of conditioned place avoidance in hamsters does not require the suprachiasmatic nucleus. Neurobiol Learn Mem 91: 81–84. 19013252 10.1016/j.nlm.2008.10.005

[6] Honma S, Honma K, Shirakawa T, Hiroshige T (1988) Rhythms in behaviors, body temperature and plasma corticosterone in SCN lesioned rats given methamphetamine. Physiol Behav 44: 247–255. 3237831 10.1016/0031-9384(88)90146-1

[7] Mendoza J, Angeles-Castellanos M, Escobar C (2005) Entrainment by a palatable meal induces food-anticipatory activity and c-Fos expression in reward-related areas of the brain. Neuroscience 133: 293–303. 15893651 10.1016/j.neuroscience.2005.01.064

[8] Yoshihara T, Honma S, Mitome M, Honma K (1997) Independence of feeding-associated circadian rhythm from light conditions and meal intervals in SCN lesioned rats. Neurosci Lett 222: 95–98. 9111737 10.1016/s0304-3940(97)13353-5

[9] Acosta-Galvan G, Yi CX, van der Vliet J, Jhamandas JH, Panula P, et al (2011) Interaction between hypothalamic dorsomedial nucleus and the suprachiasmatic nucleus determines intensity of food anticipatory behavior. Proc Natl Acad Sci U S A 108: 5813–5818. 21402951 10.1073/pnas.1015551108 PMC3078408

[10] Angeles-Castellanos M, Salgado-Delgado R, Rodriguez K, Buijs RM, Escobar C (2010) The suprachiasmatic nucleus participates in food entrainment: a lesion study. Neuroscience 165: 1115–1126. 20004704 10.1016/j.neuroscience.2009.11.061

[11] Mendoza J, Angeles-Castellanos M, Escobar C (2005) A daily palatable meal without food deprivation entrains the suprachiasmatic nucleus of rats. Eur J Neurosci 22: 2855–2862. 16324120 10.1111/j.1460-9568.2005.04461.x

[12] Gritton HJ, Kantorowski A, Sarter M, Lee TM (2012) Bidirectional interactions between circadian entrainment and cognitive performance Learning and Memory accepted for publication. 10.1101/lm.023499.111 PMC3293516 22383380

[13] Gritton HJ, Sutton BC, Martinez V, Sarter M, Lee TM (2009) Interactions between cognition and circadian rhythms: attentional demands modify circadian entrainment. Behav Neurosci 123: 937–948. 19824760 10.1037/a0017128 PMC2819151

[14] Arnold HM, Burk JA, Hodgson EM, Sarter M, Bruno JP (2002) Differential cortical acetylcholine release in rats performing a sustained attention task versus behavioral control tasks that do not explicitly tax attention. Neuroscience 114: 451–460. 12204214 10.1016/s0306-4522(02)00292-0

[15] St Peters M, Demeter E, Lustig C, Bruno JP, Sarter M (2011) Enhanced control of attention by stimulating mesolimbic-corticopetal cholinergic circuitry. J Neurosci 31: 9760–9771. 21715641 10.1523/JNEUROSCI.1902-11.2011 PMC3137238

[16] McGaughy J, Kaiser T, Sarter M (1996) Behavioral vigilance following infusions of 192 IgG-saporin into the basal forebrain: selectivity of the behavioral impairment and relation to cortical AChE-positive fiber density. Behav Neurosci 110: 247–265. 8731052 10.1037//0735-7044.110.2.247

[17] Paolone G, Lee TM, Sarter M (2012) Time to pay attention: attentional performance time-stamped prefrontal cholinergic activation, diurnality, and performance. J Neurosci 32: 12115–12128. 22933795 10.1523/JNEUROSCI.2271-12.2012 PMC3439806

[18] Bina KG, Rusak B, Semba K (1993) Localization of cholinergic neurons in the forebrain and brainstem that project to the suprachiasmatic nucleus of the hypothalamus in rat. J Comp Neurol 335: 295–307. 8227520 10.1002/cne.903350212

[19] Bina KG, Rusak B (1996) Muscarinic receptors mediate carbachol-induced phase shifts of circadian activity rhythms in Syrian hamsters. Brain Res 743: 202–211. 9017247 10.1016/s0006-8993(96)01043-8

[20] Earnest DJ, Turek FW (1983) Role for acetylcholine in mediating effects of light on reproduction. Science 219: 77–79. 6849121 10.1126/science.6849121

[21] Gillette MU, Buchanan GF, Artinian L, Hamilton SE, Nathanson NM, et al (2001) Role of the M1 receptor in regulating circadian rhythms. Life Sci 68: 2467–2472. 11392614 10.1016/s0024-3205(01)01040-2

[22] Madeira MD, Pereira PA, Silva SM, Cadete-Leite A, Paula-Barbosa MM (2004) Basal forebrain neurons modulate the synthesis and expression of neuropeptides in the rat suprachiasmatic nucleus. Neuroscience 125: 889–901. 15120850 10.1016/j.neuroscience.2004.03.005

[23] Van der Zee EA, Biemans BA, Gerkema MP, Daan S (2004) Habituation to a test apparatus during associative learning is sufficient to enhance muscarinic acetylcholine receptor-immunoreactivity in rat suprachiasmatic nucleus. J Neurosci Res 78: 508–519. 15468178 10.1002/jnr.20300

[24] Pezuk P, Mohawk JA, Yoshikawa T, Sellix MT, Menaker M (2010) Circadian organization is governed by extra-SCN pacemakers. J Biol Rhythms 25: 432–441. 21135159 10.1177/0748730410385204

[25] Stephan FK, Swann JM, Sisk CL (1979) Entrainment of circadian rhythms by feeding schedules in rats with suprachiasmatic lesions. Behav Neural Biol 25: 545–554. 464989 10.1016/s0163-1047(79)90332-7

[26] Stephan FK, Swann JM, Sisk CL (1979) Anticipation of 24-hr feeding schedules in rats with lesions of the suprachiasmatic nucleus. Behav Neural Biol 25: 346–363. 464979 10.1016/s0163-1047(79)90415-1

[27] Heckers S, Ohtake T, Wiley RG, Lappi DA, Geula C, et al (1994) Complete and selective cholinergic denervation of rat neocortex and hippocampus but not amygdala by an immunotoxin against the p75 NGF receptor. J Neurosci 14: 1271–1289. 8120624 10.1523/JNEUROSCI.14-03-01271.1994 PMC6577567

[28] Erhardt C, Galani R, Jeltsch H, Cassel JC, Klosen P, et al (2004) Modulation of photic resetting in rats by lesions of projections to the suprachiasmatic nuclei expressing p75 neurotrophin receptor. European Journal of Neuroscience 19: 1773–1788. 15078551 10.1111/j.1460-9568.2004.03281.x

[29] Scheer FA, Pirovano C, Van Someren EJ, Buijs RM (2005) Environmental light and suprachiasmatic nucleus interact in the regulation of body temperature. Neuroscience 132: 465–477. 15802197 10.1016/j.neuroscience.2004.12.012

[30] Beaule C, Amir S (2001) Photic regulation of circadian rhythms and the expression of p75 neurotrophin receptor immunoreactivity in the suprachiasmatic nucleus in rats. Brain Res 894: 301–306. 11251205 10.1016/s0006-8993(01)02021-2

[31] Haus E, Smolensky M (2006) Biological clocks and shift work: circadian dysregulation and potential long-term effects. Cancer Causes Control 17: 489–500. 16596302 10.1007/s10552-005-9015-4

[32] Reid KJ, Chang AM, Zee PC (2004) Circadian rhythm sleep disorders. Med Clin North Am 88: 631–651, viii. 10.1016/j.mcna.2004.01.010 15087208

[33] Salgado-Delgado R, Nadia S, Angeles-Castellanos M, Buijs RM, Escobar C (2010) In a rat model of night work, activity during the normal resting phase produces desynchrony in the hypothalamus. J Biol Rhythms 25: 421–431. 21135158 10.1177/0748730410383403

[34] Daan S (2000) Learning and circadian behavior. Journal of Biological Rhythms 15: 296–299. 10942260 10.1177/074873000129001396

[35] Hut RA, Van der Zee EA (2010) The cholinergic system, circadian rhythmicity, and time memory. Behav Brain Res. 10.1016/j.bbr.2010.11.039 21115064

[36] Ruby NF, Hwang CE, Wessells C, Fernandez F, Zhang P, et al (2008) Hippocampal-dependent learning requires a functional circadian system. Proc Natl Acad Sci U S A 105: 15593–15598. 18832172 10.1073/pnas.0808259105 PMC2563080

[37] Antoniadis EA, Ko CH, Ralph MR, McDonald RJ (2000) Circadian rhythms, aging and memory. Behav Brain Res 114: 221–233. 10996063 10.1016/s0166-4328(00)00290-4

[38] Mistlberger RE (1992) Anticipatory activity rhythms under daily schedules of water access in the rat. J Biol Rhythms 7: 149–160. 1611130 10.1177/074873049200700206

[39] Mistlberger RE (1993) Circadian properties of anticipatory activity to restricted water access in suprachiasmatic-ablated hamsters. Am J Physiol 264: R22–29. 8430882 10.1152/ajpregu.1993.264.1.R22

[40] Mistlberger RE, Rechtschaffen A (1985) Periodic water availability is not a potent zeitgeber for entrainment of circadian locomotor rhythms in rats. Physiol Behav 34: 17–22. 4034691 10.1016/0031-9384(85)90070-8

[41] Lee TM, Gritton HJ, Paolone G, Yan J, Hoogerwerf W, et al. Timed, sustained, attention-demanding performance reorganizes or dampens multiple circadian rhythms; 2010; Sandestin, FL. Society for Research on Biological Rhythms. 203.

[42] Abe M, Herzog ED, Yamazaki S, Straume M, Tei H, et al (2002) Circadian rhythms in isolated brain regions. J Neurosci 22: 350–356. 11756518 10.1523/JNEUROSCI.22-01-00350.2002 PMC6757616

[43] Angeles-Castellanos M, Mendoza J, Escobar C (2007) Restricted feeding schedules phase shift daily rhythms of c-Fos and protein Per1 immunoreactivity in corticolimbic regions in rats. Neuroscience 144: 344–355. 17045749 10.1016/j.neuroscience.2006.08.064

[44] Wang LM, Dragich JM, Kudo T, Odom IH, Welsh DK, et al.. (2009) Expression of the circadian clock gene Period2 in the hippocampus: possible implications for synaptic plasticity and learned behaviour. ASN Neuro 1. 10.1042/AN20090020 PMC2695588 19570032

[45] van Esseveldt KE, Lehman MN, Boer GJ (2000) The suprachiasmatic nucleus and the circadian time-keeping system revisited. Brain Res Brain Res Rev 33: 34–77. 10967353 10.1016/s0165-0173(00)00025-4

[46] Van Someren EJ (2000) Circadian and sleep disturbances in the elderly. Exp Gerontol 35: 1229–1237. 11113604 10.1016/s0531-5565(00)00191-1

[47] Bartus RT, Dean RL, 3rd, Beer B, Lippa AS (1982) The cholinergic hypothesis of geriatric memory dysfunction. Science 217: 408–414. 7046051 10.1126/science.7046051

[48] Biello SM (2009) Circadian clock resetting in the mouse changes with age. Age (Dordr). 10.1007/s11357-009-9102-7 PMC2813053 19557547

