# Supplementary figures and images for: Cognitive Performance as a Zeitgeber: Cognitive Oscillators and Cholinergic Modulation of the SCN Entrain Circadian Rhythms
Source: PLoS One. 2013 Feb 18;8(2):e56206. doi: 10.1371/journal.pone.0056206 (PMC3575350; doi:10.1371/journal.pone.0056206)

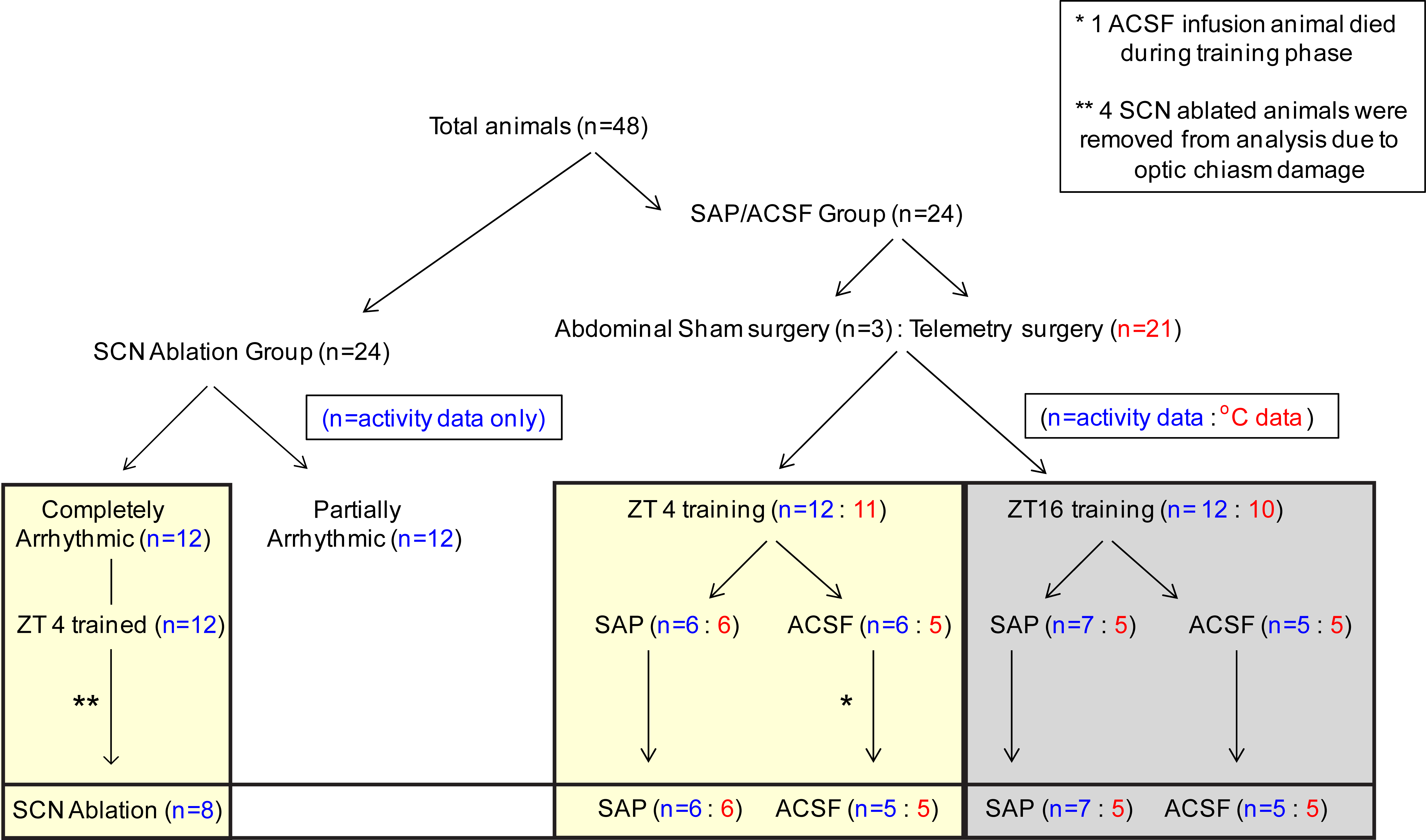

Supplement: Figure S1 — Study design and flowchart of animals used in analysis. (TIF) [file pone.0056206.s001.tif]

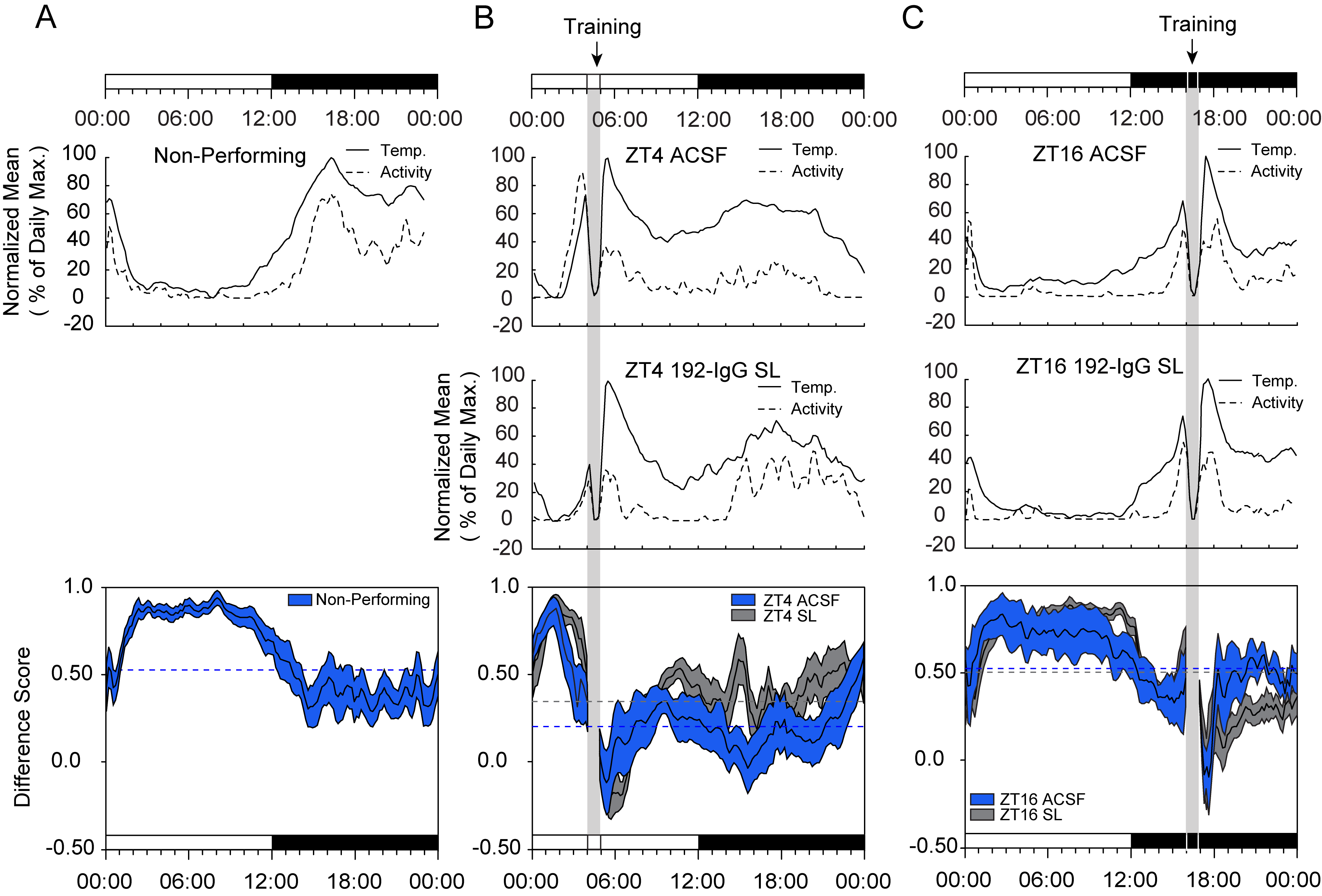

Supplement: Figure S2 — Comparisons of internal desynchrony (ID) during task training. (TIF) [file pone.0056206.s002.tif]

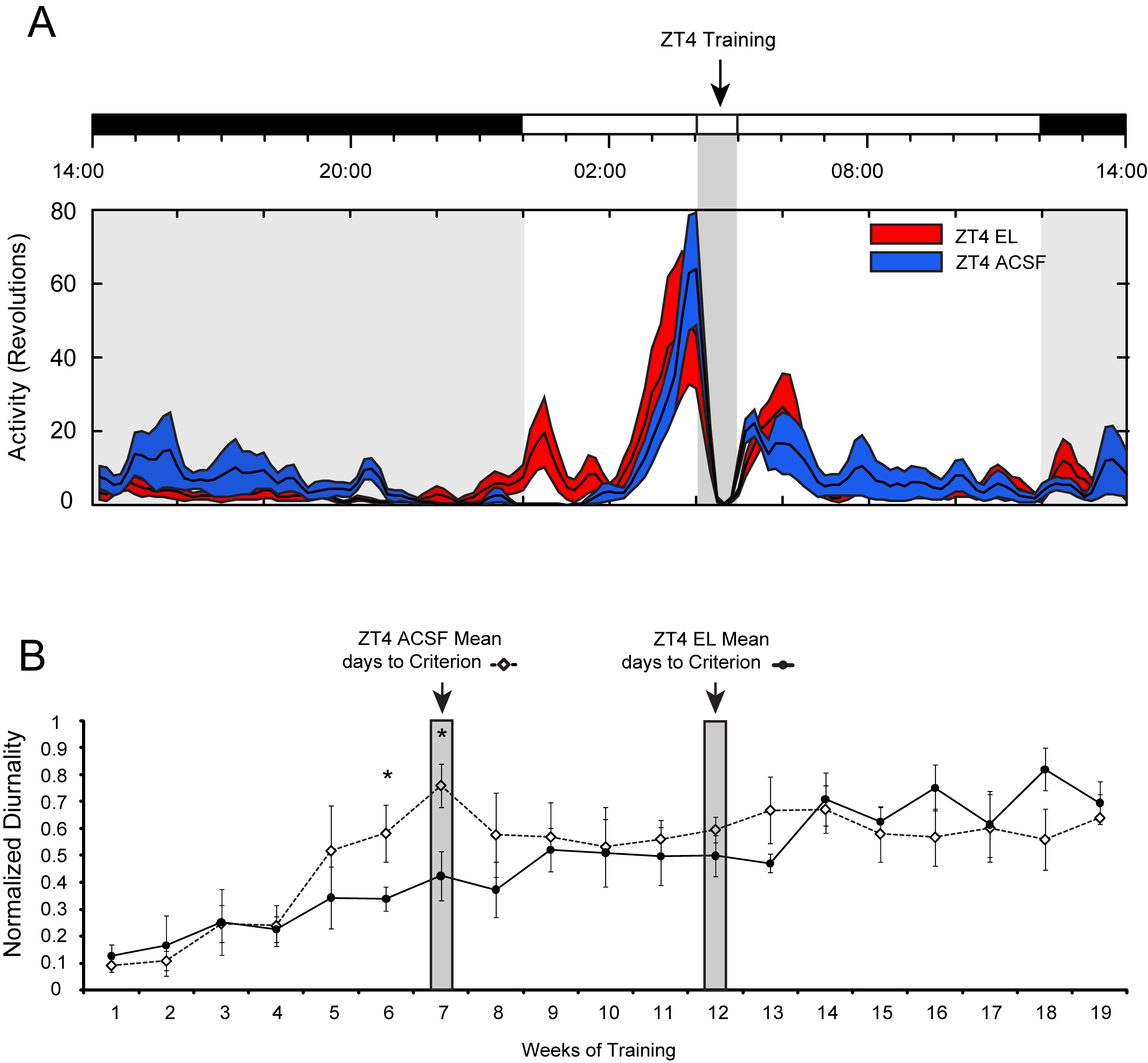

Supplement: Figure S3 — Entrainment rate during task acquisition and mean daily rhythms of SCN ablated animals. (TIF) [file pone.0056206.s003.tif]

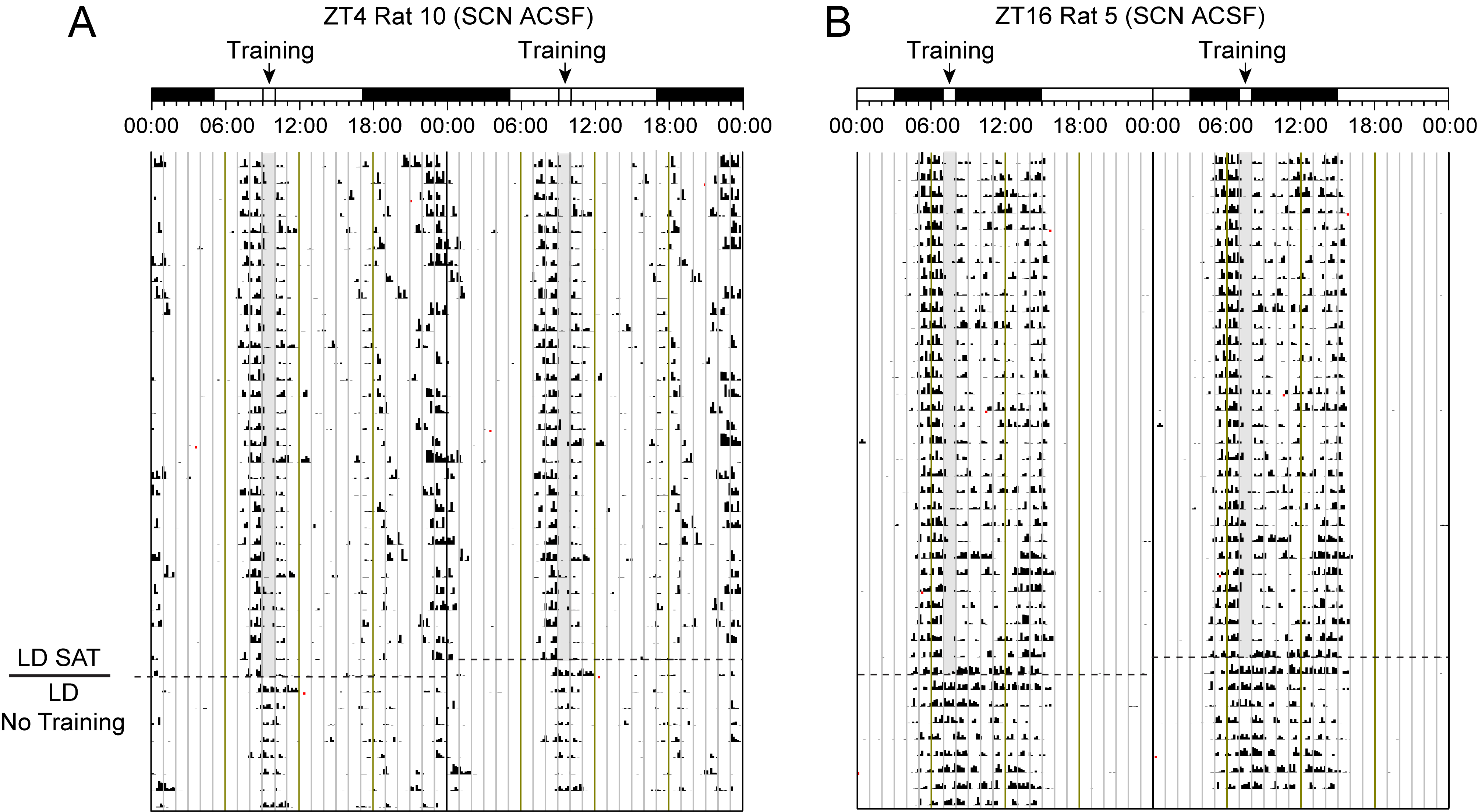

Supplement: Figure S4 — Activity records of animals removed from task in LD conditions. (TIF) [file pone.0056206.s004.tif]
