# Supplementary material for: Cognitive Performance as a Zeitgeber: Cognitive Oscillators and Cholinergic Modulation of the SCN Entrain Circadian Rhythms
Source: PLoS One. 2013 Feb 18;8(2):e56206. doi: 10.1371/journal.pone.0056206 (PMC3575350; doi:10.1371/journal.pone.0056206)
Supplement: Methods S1 — (DOCX) [file pone.0056206.s005.docx]

**Supplemental Materials and Methods**

*Subjects*

Cages were cleaned once-weekly during the animal’s training phase when absent from their home cage. Animals were weighed twice weekly during the training portion of the experiment to monitor health. Cages were lined with corn cob bedding and kept in a humidity- and temperature-controlled environment at 21±1° C.

*Surgeries and data collection*

Temperature sensitive radio transmitters (Minimitter, Inc., Bend, or) were implanted intraperitoneally (*i.p.*) in the majority of animals scheduled to undergo chemical induced lesions or vehicle infusions (*n*=21). The remaining animals underwent sham surgical procedures identical to animals given telemetry implants (*n*=3). Animals were anesthetized using isoflurane (2.5% iso, 95% oxygen) and incisions were made in the ventral abdominal area so that transmitters could be inserted into the abdominal cavity. Animals had muscle walls sutured with 4.0 chromic gut and skin incisions closed with wound clips. Clips were removed 10 days post-surgery. Animals designated to undergo SCN ablation via electrolytic lesioning did not undergo intraperitoneal surgery. Saporin-lesioned animals, and corresponding controls, underwent a second surgery at approximately six-eight weeks after arrival (four-six weeks after the initial surgery for i.p*.* telemetry implants) for subcranial infusion. 192 IgG-saporin bilateralal injections were made using a 30 gauge needle attached to a 1μL Hamilton syringe lowered once per hemisphere into the SCN (coordinates: anteroposterior: -1.0mm from bregma, lateral: ±1.1mm from the midline, dorsoventral: -8.5mm (at 6°) ventral to dura). The syringe was lowered over a period of one minute to final coordinates and remained there for two minutes prior to the injection. Following the chemical infusion, the needle remained in place for four additional minutes to allow for chemical diffusion and then slowly removed. Animals with electrolytic ablation of the SCN underwent a single surgery to ablate the SCN. A 50μm stainless steel lesioning electrode (FHC; Bowdoin, ME) with a 25μm exposed tip was lowered to four locations at the following coordinates: anteroposterior: -0.8mm and -1.2mm from bregma, lateral: ±1.1mm from the midline, dorsoventral: -8.5mm (at 6°) ventral to dura. Electrolytic lesions were produced for SCN ablation by a Grass lesion maker by passing DC current at 0.5mA for 20 seconds.

*SAT operant methods*

Animals were first trained to press a lever for a water reward in accordance with a modified fixed-ratio-1 (FR1) schedule of reinforcement. The FR1 schedule was modified in that it required animals to respond to both levers and corrected a selection bias if one existed; if rats pressed a single lever five times in a row they were required to press the opposite lever before receiving another reward. The rats were next trained to detect, discriminate, and respond to signal and non-signal events. During each session, levers extended for 4 seconds or until a lever press occurred. On signal trials, lever extension was preceded by a one-second illumination of the central panel light (signal trials), a left lever press was counted as a hit, and a right lever press was counted as a miss. In the absence of the central panel light (non-signal trials), a left lever press was counted as a false alarm, and a right lever press was counted as a correct rejection. Each correct response produced a water reward (30μL for each hit or correct rejection) and incorrect responses (miss or false alarm) were not rewarded. Half of the rats were trained in a counterbalanced fashion with the lever rules reversed. The overhead house light was off during this second phase of training, and incorrect responses resulted in trial repetition up to three times in the form of correction trials. If the rat continued to respond incorrectly after three consecutive correction trials, a forced-choice trial was initiated: a signal or non-signal event followed by extension of only the correct lever for 90 seconds or until the lever press occurred. In forced-choice signal trials, the central panel light remained illuminated for the duration of the lever extension. Each operant session consisted of 162 trials. Progression to the final training phase occurred after three consecutive days of correct responses of ≥ 69% to both signal and non-signal trials. The last phase of operant shaping, the sustained attention task (SAT), consisted of shortened signal durations (500, 50, or 25ms), shortened intertrial intervals (from the original 12±3 seconds to 9±3 seconds), and the elimination of correction and forced-choice trials. During the final stage of testing (referred to as the ‘SAT’), the house-light was illuminated requiring animals to constrain focus thus increasing attentional demand.

*Histology and immunohistochemistry*

At the completion of testing, animals were euthanized and perfused with 300mL buffered saline, followed by 300mL 4% paraformaldehyde. Brains were carefully removed and post-fixed overnight in 4% paraformaldehyde before being transferred to a 30% sucrose solution. After three days in sucrose, brains were sectioned coronally in 40µm slices with a freezing microtome (CM 2000R; Leica) and tissue sections were collected throughout the basal forebrain and SCN. Sections with SCN ablation using DC current were stained with Nissl substance only. For all other animals, alternate sections were stained for Nissl substance (cresyl violet acetate - Fisher Scientific), acetylcholinesterase (AChE), or choline acetyltransferase (ChAT). AChE staining was by a modified method of Tago et al. [1]. Briefly, after rinsing in 0.1M phosphate buffer (pH 7.4), sections were incubated in 0.1% H_2_O_2_ for 30 min, and then rinsed in 0.1M maleate buffer (pH 6.0). Sections were then immersed in a solution of: 14.70mg sodium citrate, 1.65 mg potassium ferricyanide, 7.49mg copper sulfate, and 5mg acetylthiocholine iodide in 200ml of 0.1 M maleate buffer. After rinsing with 50.0mM Tris buffer (pH 7.6), sections were incubated in a solution of 50.0mg of 3,3′-diaminobenzidine (DAB) and 375.0mg of nickel ammonium sulfate in 125.0ml of 50.0mM Tris buffer (pH 6.2). After 10 min, 10µL of 30% H_2_O_2_ was added to the sections while they continued to incubate. Staining was considered complete once cortical layering could be detected. Sections were then rinsed with 5mM Tris buffer and mounted on gelatin-coated slides and allowed to dry overnight. Following dehydration in an ascending series of alcohol rinses and clearing with xylene, slides were coverslipped with DPX.

Sections designated to undergo ChAT staining were rinsed three times in 0.1M phosphate-buffered saline (PBS, pH=7.4) for 5 min each, incubated with 0.3% peroxide for 30 minutes, followed by blocking buffer (10% goat serum in 0.1M PBS) for 60 min under conditions of constant shaking, followed by an overnight incubation with rabbit anti-CHT antibody (polyclonal goat anti-choline acetyltransferase; Millipore; Temecula, CA) diluted 1:500 in dilution buffer (0.1M PBS containing 1% goat serum and 0.1% triton X-100) at 4°C. The following day, sections were washed in wash buffer (0.1M PBS containing 0.1% triton X-100) four times for 5 min each and incubated with a biotinylated goat anti-rabbit IgG (Vectastain Elite ABC; PK-6105; Vector Laboratories; Burlingame, CA) 1:200 for 2h at 25°C. Sections were then rinsed three times for 5 minutes each in 0.2% Triton-X in 0.1M PBS and then incubated with the avidin-biotin complex (Vectastain) for 30 minutes. Following rinsing in 0.1M PBS, tissue was incubated in a peroxidase substrate solution containing 0.4% DAB and 0.19% nickel (II) chloride in 125mL 0.1M PBS. 10µL of 30% hydrogen peroxide H_2_O_2_ was added after the tissue began the incubation. Once sections showed a significant level of background stain (5-10min), sections were treated with four 5 min rinses with 0.1 M PBS and mounted on gelatin-coated slides to dry overnight. Slides were dehydrated and coverslipped as described earlier.

*Quantification of lesion effects and statistics*

Lesions using 192 IgG-saporin were designed to selectively deafferent basal forebrain cholinergic projections to the SCN. To our knowledge, only three previous studies have been published using targeted 192-IgG saporin infusions into the SCN. Studies by Beaule and Amir [2-3] used a concentration of 200ng/µl and a volume of 1µL/SCN. The most recent study used a higher concentration of 1µg/µl and a volume of 1µL/SCN [4]. Starting with these volumes and concentrations, preliminary experiments determined that smaller infusion volumes (0.4µL/SCN) were sufficient to deafferent the SCN while more selectively sparing regions outside of the SCN. In order to quantify the lesion size and location, we measured the distribution of AChE+ stained axonal fibers. 40X images were taken from two mid-SCN sections (AP-1.0 to -1.2), two sections from the central region of the anterior hypothalamus (AP-1.6 to -1.8), two sections from the medial preoptic area (AP-0.8 to -1.3), and two sections from layers 3/5 of primary motor cortex (AP-1.0 to -1.4) by an experimenter blinded to animal treatment groups using a Leica DM4000B light microscope equipped with a Spot Digital Camera using Spot Software (Diagnostics Inc., Sterling Heights, MI, USA). We used a counting grid method to determine the number of fibers in each quantified region of interest. Briefly, using Adobe Photoshop CS3 software, a grid containing 50μm squares was superimposed over each image, making a five-by-five grid (250x250μm). Crossing of the horizontal and the vertical lines of the grid were counted by two researchers, blinded to the treatment condition. Overall counts by the researchers were averaged for statistical analysis.

Images of sections (5X) containing ChAT immunopositive neurons from the five regions of the basal forebrain were taken from the medial septum (AP+0.7 to +0.2), the horizontal/vertical diagonal band (DB; AP+0.5 to -0.2), and the substantia inominata/nuc. Basalis of Meynert (SI/nBM; AP-1.1 to -1.6) by an experimenter blinded to the treatment group of each animal. The numbers of ChAT immunopositive neurons in each field of view were counted in both controls and 192 IgG-saporin injected animals. Average counts were based on two sections per region from each animal in this study. Cell counts were designed to reveal the origins of basal forebrain cholinergic projections to the SCN, but were not intended as an absolute measure of cholinergic cell loss.

The area of SCN ablation by electrolytic lesion was quantified by superimposing a stereotaxic atlas image over images of Nissl-stained sections containing the SCN ablation. Traces of the lesion outline were made using Adobe Photoshop CS3 and animals with damage to the optic chiasm were excluded from further analysis. Animals additionally had to demonstrate an arrhythmic phenotype in the presence of an LD cycle as measured by chi-square (χ^2^) periodogram analysis [5]. Analysis was performed using ClockLab (Actimetrics, Wilmette, IL) before and after SCN lesioning and was derived from 10 consecutive days of activity. Only animals showing non-significant periodicities (*p*=0.05) in the presence of an LD cycle were characterized as arrhythmic and included in subsequent analysis.

Statistical analysis of laminar and regional differences in AChE+ stained axonal fibers and ChAT cell counts were performed using *MANOVA* with region as a factor. Significant main effects were further analyzed using Tukey *post-hoc* analysis. Between-subjects effects were tested using independent t-tests for lesion condition. Alpha was set at 0.05 for the determination of statistical significance. Pearson correlation analyses were additionally done to determine relationships between days to criterion performance and cholinergic depletion based on the number of AChE-stained fibers or ChAT positive cells of the basal forebrain.

*Circadian analysis and statistics*

A repeated measure, multivariate analysis of variance (*MANOVA*) was used to assess treatment differences in the onset and offset of activities as well as the ratio of locomotor activity (LD ratio) between the light and dark-phases. Repeated within-subjects analyses of variance (*ANOVAs*) were used to determine the main effects and interactions of time of day and treatment condition for differences between activity and temperature. Significant main effects were further analyzed using *Tukey post-hoc* analysis. A probability value of *p*<0.05 was used as the criteria to determine statistical significance.

**References**

1. Tago H, Kimura H, Maeda T (1986) Visualization of detailed acetylcholinesterase fiber and neuron staining in rat brain by a sensitive histochemical procedure. J Histochem Cytochem 34: 1431-1438.

2. Beaule C, Amir S (2001) Photic regulation of circadian rhythms and the expression of p75 neurotrophin receptor immunoreactivity in the suprachiasmatic nucleus in rats. Brain Res 894: 301-306.

3. Beaule C, Amir S (2002) Effect of 192 IgG-saporin on circadian activity rhythms, expression of P75 neurotrophin receptors, calbindin-D28K, and light-induced Fos in the suprachiasmatic nucleus in rats. Exp Neurol 176: 377-389.

4. Erhardt C, Galani R, Jeltsch H, Cassel JC, Klosen P, et al. (2004) Modulation of photic resetting in rats by lesions of projections to the suprachiasmatic nuclei expressing p75 neurotrophin receptor. European Journal of Neuroscience 19: 1773-1788.

5. Sokolove PG, Bushell WN (1978) The chi square periodogram: its utility for analysis of circadian rhythms. J Theor Biol 72: 131-160.
