# Supplementary material for: Cognitive Performance as a Zeitgeber: Cognitive Oscillators and Cholinergic Modulation of the SCN Entrain Circadian Rhythms
Source: PLoS One. 2013 Feb 18;8(2):e56206. doi: 10.1371/journal.pone.0056206 (PMC3575350; doi:10.1371/journal.pone.0056206)
Supplement: Results S1 — (DOCX) [file pone.0056206.s006.docx]

**Supplemental Results**

*Cognitive training of nocturnal rodents during the light-phase promotes internal desynchrony*

As described previously, temperature rhythms and activity rhythms in task-performing animals do not share the same phase relationship. We compared core body temperature to activity for all subjects used in this study prior to training to establish a baseline (non-performing: NP) for subsequent comparisons of relative internal desynchrony (***supplemental figure S2A***). In order to quantify this, we normalized body temperature and activity to daily minimums and maximums for each animal and quantified difference scores bin by bin across the light-cycle and the dark-cycle. Comparisons of treatment groups (***supplemental figure S2B***: ***ZT4****;* ***supplemental figure S2C****:* ***ZT16***) revealed an effect of training group for the lights-on phase (*F*(3,17) = 18.497, *p*<0.001) and lights-off phase (*F*(3,17) = 3.242, *p*<0.048) of training. Analysis during the lights-on phase found significant higher levels of ID between ZT4 controls and ZT16 controls (*p*<0.001) and ZT16 SL (*p*<0.001), but not ZT4 SL (*p*=0.446). Analysis of ID during the dark-phase also found significantly greater levels of ID in ZT4 controls relative to all other treatment groups (ZT16 controls, *p*=0.015; ZT16 SL, *p*=0.029, and ZT4 SL, *p*=0.022). This effect was limited to ZT4 control animals as ZT4 SL animals did not differ from either ZT16 training group during the dark-phase. This finding suggests that ZT4 SL animals maintain a high nighttime level of activity, comparable to animals training at ZT16, and a substantially lower level of activity during the lights-on phase than ZT4 control animals. We also compared overall ID based on daily means (*difference score: dashed-lines,* ***supplemental figure S2:*** *bottom*) for all subjects relative to their baseline (non-performing) ID ratios. Difference score values approaching 1 represent periods of greatest coherence (see figure legend). There was a significant effect of treatment group on relative ID (*F*(4,37) = 11.887, *p*<0.001) and *post-hoc* analysis revealed both ZT4 groups showed significantly more daily ID relative to their baseline condition (ZT4 controls, *p*<0.001; ZT4 SL, *p*=0.043) that was not observed in the ZT16 training group. ZT4 controls showed enhanced daily ID relative to both ZT16 training groups (both, *p*<0.001) that was not significant for ZT4 SL animals (ZT16 controls, *p*=0.101; ZT16 SL, *p*=0.089).

*Ablation of the SCN impairs SAT task acquisition*

Initial analysis found no significant differences in overall hit rate (average of all signal durations (*F*(4,26) = 1.891, *p*=0.142), number of correct rejections (*F*(4,26) = 1.702, *p*=0.180), or omissions (*F*(4,26) = 0.515, *p*=0.726) across all treatment groups.

**Supplemental figure S3B** plots normalized diurnality during the 19 weeks of training for ZT4 controls and ZT4 EL training groups. A value of 0 corresponds to minimal diurnality across the training window and a value of 1 represents the maximal level of diurnality. For example, a value of 1 could only be achieved if all animals from the same treatment group showed their maximal diurnality during the same week of the training period. We found a significant interaction between treatment group and diurnality over the first seven weeks of training, (*F*(6,66) = 4.058, *p*=0.004) that did not exist for the remainder of task training (*F*(11,121) = 1.245, *p*=0.277). Control animals show a more rapid change from their baseline LD ratio that became maximally diurnal in the week that the group reaches criterion performance. SCN ablated animals did not show peak diurnality until the final weeks of training revealing that an ability of oscillators to time daily training may facilitate task acquisition.

In addition to delayed acquisition, we noted that ZT4 EL animals took substantially longer to establish a synchronized relationship to the time of daily SAT training (*see* ***supplemental figure S3B***). Because SCN ablated animals acquire the task more slowly, but eventually show the most daytime activity, we examined how task related activity changes over the course of task acquisition for these animals. Our results revealed that SCN ablated animals did not show peak diurnality until the final weeks of training suggesting that timing of daily training and associated synchronization may additionally serve as an essential component of task acquisition.
